# Haploinsufficiency in the *ANKS1B* gene encoding AIDA-1 leads to a neurodevelopmental syndrome

**DOI:** 10.1038/s41467-019-11437-w

**Published:** 2019-08-06

**Authors:** Abigail U. Carbonell, Chang Hoon Cho, Jaafar O. Tindi, Pamela A. Counts, Juliana C. Bates, Hediye Erdjument-Bromage, Svetlana Cvejic, Alana Iaboni, Ifat Kvint, Jenny Rosensaft, Ehud Banne, Evdokia Anagnostou, Thomas A. Neubert, Stephen W. Scherer, Sophie Molholm, Bryen A. Jordan

**Affiliations:** 1 Dominick P. Purpura Department of Neuroscience, Albert Einstein College of Medicine, Bronx, 10461 NY USA; 2 Department of Pediatrics, Albert Einstein College of Medicine, Bronx, 10461 NY USA; 3 Department of Cell Biology and Kimmel Center for Biology and Medicine of the Skirball Institute, New York University School of Medicine, New York, 10016 NY USA; 4 Autism Research Centre, Bloorview Research Institute, Holland Bloorview Kids Rehabilitation Hospital, Toronto, M46 1R8 ON Canada; 5 Pediatric Neurology Clinic, Kaplan Medical Center, Hebrew University Hadassah Medical School, Rehovot, 76100 Israel; 6 Genetics Institute, Kaplan Medical Center, Hebrew University Hadassah Medical School, Rehovot, 76100 Israel; 7 Department of Pharmacology, New York University School of Medicine, New York, 10016 NY USA; 8 Centre for Applied Genomics and McLaughlin Centre, Hospital for Sick Children and University of Toronto, Toronto, M56 0A4 ON Canada; 9 Department of Psychiatry and Behavioral Sciences, Albert Einstein College of Medicine, Bronx, 10461 NY USA

**Keywords:** Proteomic analysis, Autism spectrum disorders, Developmental disorders, Genetics of the nervous system

## Abstract

Neurodevelopmental disorders, including autism spectrum disorder, have complex polygenic etiologies. Single-gene mutations in patients can help define genetic factors and molecular mechanisms underlying neurodevelopmental disorders. Here we describe individuals with monogenic heterozygous microdeletions in *ANKS1B*, a predicted risk gene for autism and neuropsychiatric diseases. Affected individuals present with a spectrum of neurodevelopmental phenotypes, including autism, attention-deficit hyperactivity disorder, and speech and motor deficits. Neurons generated from patient-derived induced pluripotent stem cells demonstrate loss of the *ANKS1B*-encoded protein AIDA-1, a brain-specific protein highly enriched at neuronal synapses. A transgenic mouse model of *Anks1b* haploinsufficiency recapitulates a range of patient phenotypes, including social deficits, hyperactivity, and sensorimotor dysfunction. Identification of the AIDA-1 interactome using quantitative proteomics reveals protein networks involved in synaptic function and the etiology of neurodevelopmental disorders. Our findings formalize a link between the synaptic protein AIDA-1 and a rare, previously undefined genetic disease we term *ANKS1B* haploinsufficiency syndrome.

## Abstract (web summary)

Understanding of the genetic factors and molecular mechanisms underlying neurodevelopmental disorders remains incomplete. In this study, authors show that microdeletions in the gene ANKS1B lead to loss of the neuronal synapse-enriched protein AIDA-1 and to a novel neurodevelopmental syndrome

## Introduction

Neurodevelopmental disorders, including autism spectrum disorder (ASD), attention-deficit/hyperactivity disorder (ADHD), and communication and motor disorders, have complex polygenic etiologies. These disorders are highly comorbid and share hereditary risk factors, suggesting that perturbations in pathways regulating brain development can result in a range of neurodevelopmental phenotypes^1^. In current models of autism heritability, common and rare variants interact with environmental factors to confer genetic risk^2^. Although de novo variants are often used to causally link individual genes to autism, inherited mutations in highly conserved genes also appear at higher rates in neurodevelopmental disorders and are noted to display variable penetrance^3^. Ultra-rare copy-number variations (CNVs) covering >1 kilobase are predicted to make a sizable contribution to risk for neurodevelopmental disorders^4^.

Recent analyses of genetic risk for autism identified *ANKS1B* as a target gene due to rare genetic variants found in ASD^5–7^, predicted participation in gene networks dysregulated in ASD^8,9^, and salience in bioinformatic analyses of mouse phenomics^10^. Genomic and transcriptomic association studies of neuropsychiatric disorders implicate *ANKS1B* in obsessive-compulsive disorder^11,12^, mood disorders^13,14^, and schizophrenia^15–18^. *ANKS1B* is a large ~1.3 megabase gene located on human chromosome 12q23.1 that encodes AIDA-1 (APP intracellular domain associated 1), a protein initially suggested to regulate γ-secretase processing of amyloid precursor protein (APP)^19^. We have shown that AIDA-1 is highly expressed in the brain, where it is enriched in hippocampal and cerebellar regions^20^ and is one of the most abundant proteins at neuronal synapses^21,22^. AIDA-1 is specifically enriched at postsynaptic densities (PSDs), where it binds to N-methyl-d-aspartate receptors (NMDARs) and the adaptor protein PSD95^23^. Neuronal activity causes rapid translocation of AIDA-1 into the nucleus, resulting in changes in Cajal body stability and nucleolar morphology^23,24^. Recently, we found that postnatal deletion of AIDA-1 from the forebrain led to reduced synaptic expression of the NMDAR subunit GluN2B and impaired NMDA-dependent long-term potentiation and long-term depression in the hippocampus^25^.

Despite the literature suggesting an association between *ANKS1B* and neurodevelopmental disease, no patients with confirmed loss of function in *ANKS1B* have been previously identified. Here, we describe monogenic CNVs in *ANKS1B* in individuals that display a spectrum of neurodevelopmental phenotypes, including ASD, ADHD, and speech and motor deficits. A newly generated mouse model of *ANKS1B* haploinsufficiency syndrome exhibits behavioral correlates of the phenotypes observed in probands. Along with new evidence that AIDA-1 interacts with multiple regulators of neural development, our findings demonstrate that haploinsufficiency of *ANKS1B* leads to a previously uncharacterized neurodevelopmental syndrome.

## Results

### *ANKS1B* deletion probands have neurodevelopmental disorders

We identified two families (EIN-1 and EIN-2) harboring monogenic microdeletions in *ANKS1B* who had been referred for medical genetic evaluation due to various neurodevelopmental disorders, including autism, ADHD, speech apraxia, and motor delays (Table 1). We performed extensive neuropsychological testing and clinical interviews on all affected individuals from families EIN-1 and EIN-2 (Supplementary Data 1). Affected individuals displayed a pattern of developmental delays, oculomotor and oromotor irregularities, dysmetria, impaired fine-motor dexterity, and problems with balance and gait. Subjects had a normal Full Scale Intelligence Quotient (FSIQ standard score 85–115), except for a female proband with speech apraxia (EIN-2-1) whose composite FSIQ (standard score = 81) was reduced by a verbal comprehension index (VCI) standard score of 73. Verbal impairments were more severe in female children (EIN-1-2 and EIN-2-1), consistent with their prior diagnosis of speech apraxia, and manifested in lower scores in VCI, expressive language, receptive language, and verbal memory compared to males. On a test of fine-motor dexterity and psychomotor speed, all children performed at least two standard deviations below the mean with their dominant hand (*z*-score –2.22 to –2.89), whereas performance with the non-dominant hand was relatively spared. Deficits in oculomotor and fine-motor coordination contributed to poor or borderline performance in tasks requiring visual-motor integration. Consistent problems with speech and motor control contributed to variable performance on tests of attention and executive function (Supplementary Data 1).

**Table 1:** *ANKS1B* microdeletion probands display a spectrum of neurodevelopmental phenotypes

ID	EIN-1-1	EIN-1-2	EIN-1-3	EIN-2-1	TOR-1	TOR-2	GEN-1	DEC-1	DEC-2
Sex	46XY	46XX	46XY	46XX	46XY	46XY	46XX	46XY	46XX
Age	11	6	3	5	7	2	3	7	8
Variant/deletion (GRCh37/hg19)	12:99609297-99986120	12:99609297-99986120	12:99609297-99986120	12:99930346-100284065	12:100099666-100230043	12:100112323-100192972	12:99945859-100303670	12:99727586-100152135	12:99994978-100105971
Variant detection	CMA/WES	CMA	CMA	CMA/WES	WGS	WGS	CMA/WES	CMA	CMA
Inheritance	Paternal	Paternal	Paternal	Paternal	Paternal	Maternal	Unknown	Maternal	Paternal
Ethnicity	Caucasian	Caucasian	Caucasian	Caucasian	Caucasian	South Asian	Caucasian	Caucasian	Caucasian
Variant size	377 kb	377 kb	377 kb	354 kb	130 kb	80 kb	358 kb	425 kb	111 kb
Allele	Heterozygous	Heterozygous	Heterozygous	Heterozygous	Heterozygous	Heterozygous	Heterozygous	Heterozygous	Heterozygous
Craniofacial dysmorphism	Brachycephalic, round facies, prominent brow; midface hypoplasia	Brachycephalic, round facies, midface hypoplasia	Brachycephalic, midface hypoplasia, positional plagiocephaly	Round facies, prominent metopic suture, small right ear with slight cupping	None reported	None reported	Round facies, occipital flattening, upslanting palpebral fissure, enlarged tongue	Hypertelorism, preauricular pit, short nose, thick nasal alae	Synophrys, downslanting palpebral fissure, anteverted nares, short philtrum
Head circumference	92nd percentile	90th percentile	60th percentile	<3rd percentile	91st percentile	95th percentile	Not reported	50th percentile	2nd percentile
Intellectual disability	No	No	No	No	Yes	Yes	Yes	No	No
Developmental delay	Yes	Yes	Yes	Yes	No	No	Yes	No	Yes
ASD	Yes	No	No	No	Yes	Yes	Yes	No	Yes
ADHD	Yes	Yes	No	No	No	No	No	Yes	Yes
Other psychiatric	Aggressive behavior, dysgraphia, self-soothing behaviors	Academic difficulty	Head banging	None	None	None	Poor play, eye contact, and name response; no imitation or pattern matching	None	Low frustration tolerance, reactive anger outbursts
Speech delay	Yes	Yes	Yes	Yes	Yes	Yes	Yes	No	Yes
Speech apraxia	No	Yes	No	Yes	No	No	Yes	No	No
Motor delay	Yes	Yes	Yes	Yes	No	Yes	Yes	No	No
Motor dyspraxia	No	Yes	Yes	No	No	No	Yes	No	No
Other neurologic	Vocal/motor Tourette’s, hypersensitive sensory processing deficit	Encopresis	Plagiocephaly, sleep disturbance, congenital tortocollis, myoclonic jerks, hypertonicity	None	None	None	None	None	None
MRI	None	Hyperintensities in caudate nucleus, thin corpus callosum	Normal	None	None	Hyperintensity in left periventricular white matter, thin corpus callosum body	Dysgenesis of corpus callosum, absent splenium	None	Enlarged ventricles, thin corpus callosum
EEG	Normal	None	Normal	None	None	Normal	Normal	None	None
Other	Astigmatism, high palate	Astigmatism, high palate, asthma	Ear tubes, high arched palate, frenulectomy,	Choanal atresia, pulmonary artery stenosis, atrial shunting	None reported	None reported	None reported	Brachydactyly, short phalanx of finger, short toe	Specialized schooling required

Patients harboring heterozygous and monogenic microdeletions in the *ANKS1B* gene were identified at Albert Einstein College of Medicine (EIN), the Autism Speaks MSSNG project at the University of Toronto (TOR), the DECIPHER project (DEC), and the GeneMatcher online resource (GEN). Several individuals were diagnosed with autism (ASD) and ADHD, and most display speech and motor phenotypes, including delayed achievement of developmental milestones, speech apraxia, and motor dyspraxia. Craniofacial dysmorphisms and abnormal MRI findings were also reported in several families. Two probands had head circumference meeting criteria for microcephaly (<3rd percentile). Variant detection refers to the method employed to identify the deletions

*CMA*  chromosomal microarray analysis, *WGS* whole-genome sequencing, *WES*  Whole-exome sequencing

In parent-rated assessments, both sets of parents uniformly endorsed low to average social skills in affected children (Supplementary Data 1). While neither of the affected female children met criteria for a social communication disorder, they showed deficits in affect recognition and theory of mind tasks. Affected children inherited the *ANKS1B* microdeletion from a parent previously reported to have a mild (EIN-1-4) or normal (EIN 2-2) phenotype. However, we found that the affected parent in EIN-1, who had reported childhood motor delays, also displayed oculomotor, fine motor, visual motor, and gross motor deficits. The affected father in EIN-2, who had a previous diagnosis of ADHD, displayed impaired oculomotor control, visual-motor integration, and gross motor coordination (Supplementary Data 1). Moreover, a new diagnosis of autism was issued for one affected parent (EIN-1-4) at the conclusion of the study.

*ANKS1B* copy-number variations have not previously been associated with a genetic syndrome. We therefore searched genotype-phenotype databases for individuals harboring similar CNVs. We identified ten additional probands in North America, Europe, and the Middle East harboring monogenic microdeletions in *ANKS1B* through the Autism Speaks MSSNG project at the University of Toronto (TOR-1 and TOR-2), the DECIPHER project^26^ (DEC-1,2,4,5,8,9,12), and the GeneMatcher online resource^27^ (GEN-1) (Table 1 and Supplementary Data 2). We obtained clinical evaluations for five probands with monogenic deletions, as well as for four probands with CNVs in additional genes (Supplementary Data 2). Deletions in all individuals were identified during clinical testing through chromosomal microarray except for EIN-1-4 and EIN-2-2, which were confirmed by FISH, and TOR-1 and TOR-2, which were identified through whole-genome sequencing. All affected individuals were heterozygous for the *ANKS1B* microdeletion, and most deletions were inherited (Table 1 and Supplementary Data 2). We performed additional whole-exome sequencing in trios (proband and both parents) from the EIN-1 and EIN-2 families. No loss-of-function variants annotated with neurodevelopmental disorders in ClinVar^28^ were identified specifically in affected individuals (Supplementary Data 3). These results suggest that the monogenic deletions in *ANKS1B* underlie the phenotypes identified in EIN-1 and EIN-2.

*ANKS1B* deletion in these additional probands resulted in neurodevelopmental phenotypes consistent with our findings in the first two families (Table 1). ASD and ADHD were common diagnoses, along with speech and motor dyspraxia. Developmental delays, including delayed achievement of speech and motor milestones, were prevalent. Although intellectual disability was a variable finding, low IQ and global developmental delay were noted in a few patients. Several patients showed abnormal magnetic resonance imaging (MRI) findings, including T2 hyperintensities in various regions and dysgenesis or thinning of the corpus callosum. Craniofacial dysmorphisms were also reported in several families, although specific features were not consistent among probands. Additional individuals harboring monogenic deletions in *ANKS1B*, as well as those with multiple CNVs affecting other genes (Supplementary Data 2), displayed similar neurodevelopmental phenotypes. All individuals identified in this study had received a clinical diagnosis before the age of 18.

The rarity of patients with CNVs in this gene is likely due to intolerance of *ANKS1B* loss of function in the population. Scarcity of *ANKS1B* variants was a factor in its identification as a brain-enriched gene linked to autism^6^. Using a predictive model of haploinsufficiency, *ANKS1B* is predicted to cause dominantly-inherited disease^29^. From exome sequencing data obtained by the Exome Aggregation Consortium (ExAC), *ANKS1B* has a computed pLI (probability of loss-of-function intolerance) of 0.99, indicating that fewer than 10% of the expected protein-truncating variants are observed for the gene^30^. Furthermore, analysis of local missense constraint identifies two discrete regions in *ANKS1B* with significantly less missense variation than expected: the seven N-terminal exons (regional missense constraint = 0.451, likelihood ratio test, *p* = 2.29e-7), and the nine C-terminal exons (regional missense constraint = 0.412, likelihood ratio test, *p* = 1.65e-8)^31^. These results indicate that variation in *ANKS1B*, including the N-terminal region affected by most of the microdeletions we identified, is strongly selected against in the population.

### *ANKS1B* deletions lead to loss of AIDA-1 in patient neurons

**Fig. 1:**
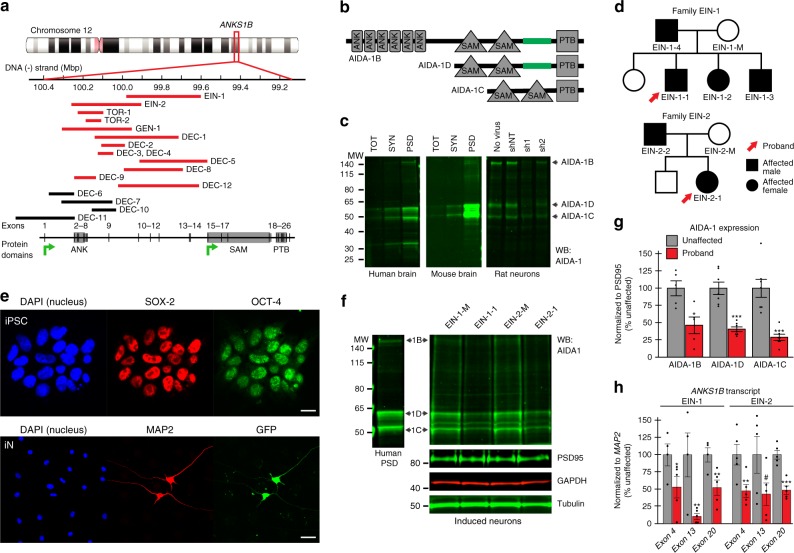
*ANKS1B* microdeletions reduce AIDA-1 expression in patient-derived neurons. **a** Monogenic microdeletions (red bars) in patients span multiple exons in the *ANKS1B* gene. Black bars represent patients with additional CNVs in other genes. Green arrows represent putative transcription start sites. **b** *ANKS1B* encodes AIDA-1B, AIDA-1D, and AIDA-1C protein isoforms. **c** (Left) Western blots show that all three major AIDA-1 isoforms are expressed in total lysate (TOT) and enriched in synaptic (SYN) and postsynaptic density (PSD) fractions in postmortem human and mouse brain (5 μg lysate). (Right) Lentivirus-mediated knockdown of AIDA-1 in primary rat neurons (DIV 14-21) using two different shRNAs (sh1 and sh2 = AIDA-1 specific; shNT = scrambled control shRNA) confirms antibody specificity (20 μg lysate). **d** Pedigree of EIN-1 and EIN-2 families. **e** (Top) IPSCs generated from probands (EIN-1-1 and EIN-2-1) and unaffected mothers (EIN-1-M and EIN-2-M) express Oct-4 and Sox-2 pluripotency markers. (Bottom) Induced neurons (iNs) generated from forced *NGN2* expression (GFP) in iPSCs show typical neuronal morphology and express the mature neuronal marker MAP2. Nuclei from non-neuronal cells are co-cultured rat astrocytes added to improve neuronal viability and maturation. Scale bars = 10 μm. **f** Western blots of AIDA-1 and PSD95 show that AIDA-1 isoforms are significantly reduced in probands EIN-1-1 and EIN-2-1 compared to unaffected mothers EIN-1-M and EIN-2-M (10 μg lysate). **g** Quantitation of AIDA-1 expression normalized to neuronal marker PSD95 from Family EIN-2. *N* = 5–7 biological replicates. Bar graphs show mean ± SEM, two-sided Student’s *t*-test, *p < 0.05 ***p < 0.001. **h** RT-qPCR of exons 4, 13, and 20 spanning the *ANKS1B* gene and normalized to neuronal marker *MAP2* are reduced in probands from both families. *N* = 4–5 biological replicates for each family. Bar graphs show mean ± SEM, one-sided Student’s *t*-test, *p < 0.05, **p < 0.01, ***p < 0.001, ^#^*p* = 0.052

*ANKS1B* microdeletions in patients varied in size between 80-425 kilobases and targeted multiple exons in the 5′ region of the gene, predicting loss of AIDA-1 transcript and protein (Fig. 1a). However, *ANKS1B* is a complex gene encoding more than 30 transcripts^32^, some of which reside outside the deletions. Assignation of exons is variable between genome repositories (NCBI, ENSEMBL), but all full-length *ANKS1B* transcripts encode a protein containing six N-terminal ankyrin repeats (ANK), two sterile alpha motif (SAM) domains, and a C-terminal phosphotyrosine binding (PTB) domain (AIDA-1B), while shorter isoforms lack the ankyrin repeats (AIDA-1D and AIDA-1C) (Fig. 1b). Western blots of postmortem human brain and mouse brain tissue show that in both species, AIDA-1B, AIDA-1D, and AIDA-1C are the main isoforms expressed in whole lysates and enriched in synaptic (SYN) and postsynaptic density (PSD) fractions. Knockdown of AIDA-1 expression in primary rat neurons using short-hairpin RNA (shRNA)^25^ confirms the specificity of AIDA-1 antibodies in human lysates since rodent and human AIDA-1 sequences are identical (Fig. 1c).

To determine how human *ANKS1B* microdeletions affect AIDA-1 expression, we generated induced pluripotent stem cells (iPSCs) using peripheral mononucleated blood cells from probands and unaffected mothers in families EIN-1 and EIN-2 (Fig. 1d) through forced expression of transcription factors using Sendai virus^33^. Human-derived iPSCs were positive for pluripotency markers Oct-4 and Sox-2 by immunostaining (Fig. 1e). As AIDA-1 is enriched in neuronal synapses, we generated neurons by overexpressing the neuronal transcription factor neurogenin-2 (*NGN2*)^34^. Differentiation of induced neurons (iNs) was confirmed by observed morphology and by immunostaining for the neuronal marker MAP2. Western blots of iNs showed that heterozygous *ANKS1B* deletion in the EIN-2 proband reduced AIDA-1 expression compared to the unaffected mother (Fig. 1f, g). Similar results were obtained for the EIN-1 family (Supplementary Data 4). While we had expected a selective reduction in the full-length AIDA-1B isoform, our results show that deletions upstream of AIDA-1D and AIDA-1C transcripts similarly reduce expression of these shorter isoforms. Loss of major protein products suggests a model of genetic haploinsufficiency, but microdeletions could also result in abnormal RNAs and truncated polypeptides that contribute to disease. To address this possibility, we performed reverse transcription quantitative polymerase chain reaction (RT-qPCR) for exons along the *ANKS1B* gene to identify abnormally generated transcripts. In both EIN-1 and EIN-2, families that contain distinct microdeletions, exons in N-terminal, middle, and C-terminal regions were downregulated in proband iNs (Fig. 1h). In addition, we performed western blots of iNs using AIDA-1 antibodies to other regions of the protein and observed similar changes (Supplementary Fig. 1). Altogether, these results strongly implicate genetic haploinsufficiency in disease etiology.

### *ANKS1B* haploinsufficiency mouse model shows loss of AIDA-1

**Fig. 2:**
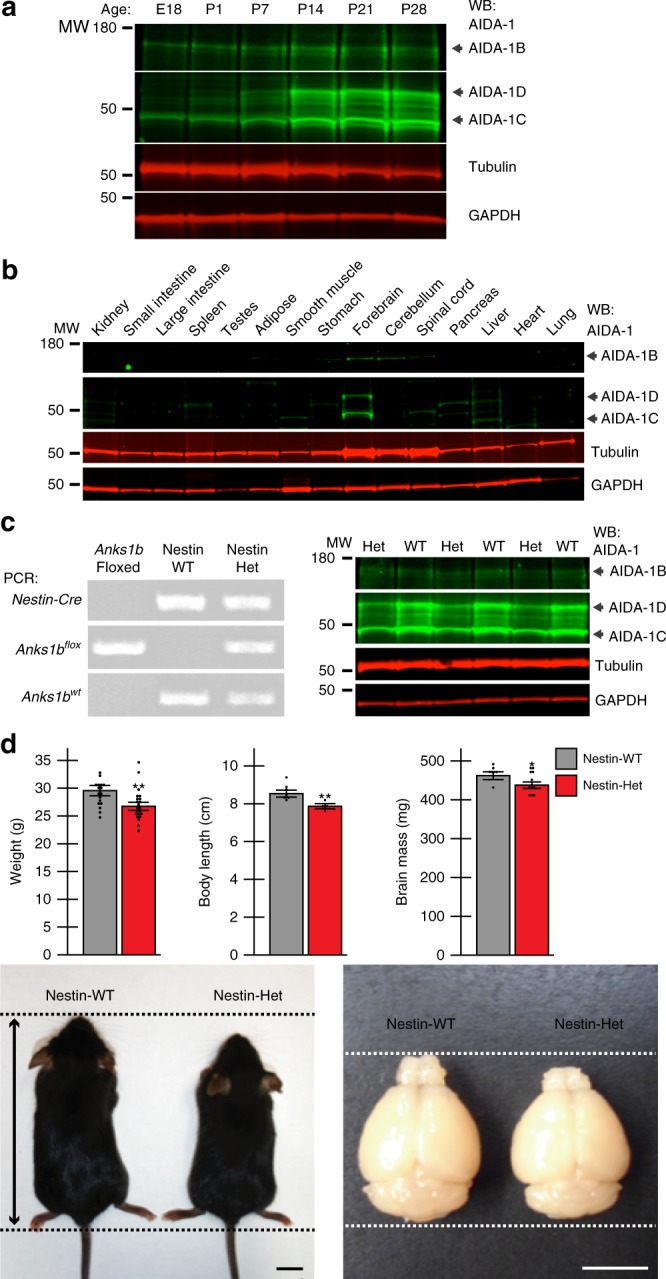
Heterozygous *Anks1b* knockout mice are viable and show reduced AIDA-1 expression. **a** Western blots show AIDA-1B is expressed in mouse brain tissue from embryonic development to adulthood. AIDA-1D and 1C expression increases until reaching stable levels in the adult brain. Tubulin and GAPDH levels are shown as loading controls (20 μg lysate). E = embryonic day, P = postnatal day. **b** AIDA-1 isoforms are selectively expressed in whole mouse brain and cerebellum (20 μg lysate). **c** (Left) PCR genotyping to identify Nestin-Het mice. (Right) Western blots show reduced expression of AIDA-1 isoforms in Nestin-Het mice (20 μg lysate). **d** Male Nestin-Het mice show decreased total weight (26.7 ± 0.6 g, mean ± SEM) compared to Nestin-WT controls (29.5 ± 0.8 g); *N* = 41 mice. Body length was also reduced (7.88 ± 0.09 cm) compared to controls (8.55 ± 0.14 cm); *N* = 13 mice, scale bar in representative image = 1 cm. Brain mass was also reduced (Nestin-Het = 442.7 ± 8.5 mg, Nestin-WT = 467.5 ± 6.2 mg); *N* = 19 mice, scale bar in representative image = 0.5 cm. Bar graphs show mean ± SEM, two-sided Student’s *t*-test, *p < 0.05, **p < 0.01. No statistically significant differences were observed in female mice (Supplementary Data 4)

Probands with *ANKS1B* microdeletions exhibit neurodevelopmental disorders in childhood, suggesting dysregulation of early neural development. However, we had shown that shorter AIDA-1 isoforms are expressed postnatally in mice, reaching detectable levels 7–14 days after birth^25^. Western blots of mouse brain across development demonstrate that unlike AIDA-1D and AIDA-1C, the large AIDA-1B isoform is expressed from embryonic to adult stages (Fig. 2a and Supplementary Fig. 2a). Gene expression databases and postmortem human tissue analyses^35^ show that *ANKS1B* expression is highly brain-selective. In mouse tissue, we found that AIDA-1 isoforms are exclusively detected in brain, with the cerebellum expressing only the larger AIDA-1B isoform (Fig. 2b). Given the embryonic and neural expression pattern of AIDA-1, we used a transgenic *Nestin-Cre* mouse line to delete *Anks1b* from the developing central and peripheral nervous system starting at embryonic day 11^36^. Crossing *Nestin-Cre* mice to the *Anks1b* floxed mouse line previously developed^25^ revealed that homozygous AIDA-1 *Nestin-Cre* knockout mice (*Nestin-Cre;Anks1b*^*flox/flox*^) do not survive beyond postnatal day 0, highlighting a critical role for neural *Anks1b* in development. This observation of embryonic lethality is consistent with predicted loss-of-function intolerance in *ANKS1B*^30^ and the lack of identified patients homozygous for *ANKS1B* deletions.

Unlike homozygous *Nestin-Cre* knockouts, heterozygous *Anks1b* mice (*Nestin-Cre;Anks1b*^*wt/flox*^, referred to here as Nestin-Het mice) were viable (Fig. 2c). Given that behavioral phenotypes have been reported in the *Nestin-Cre* transgenic line^37^, we used *Nestin-Cre;Anks1b*^*wt/wt*^ littermates (Nestin-WT mice) as controls for Nestin-Het mice in all experiments. Heterozygous *Anks1b* deletion resulted in a ~50% reduction in AIDA-1 isoforms by western blot (Fig. 2c and Supplementary Fig. 2b, c) and reduced expression across *Anks1b* exons by RT-qPCR similar to the changes observed in *ANKS1B* microdeletion probands (Supplementary Fig. 2d). Gross characterization of Nestin-Het mice revealed small but significant sex-specific differences, with a decrease in weight, body length, and brain mass in male mice that was not significant in females (Fig. 2d and Supplementary Data 4). Since AIDA-1 is enriched in the hippocampus and cerebellum, we performed a histological analysis of morphology in these brain regions. We found no significant changes in the size of these regions and no sex-dependent differences by 2-way ANOVA (Supplementary Fig. 2e and Supplementary Data 4).

### *Anks1b* mouse model recapitulates patient phenotypes

**Fig. 3:**
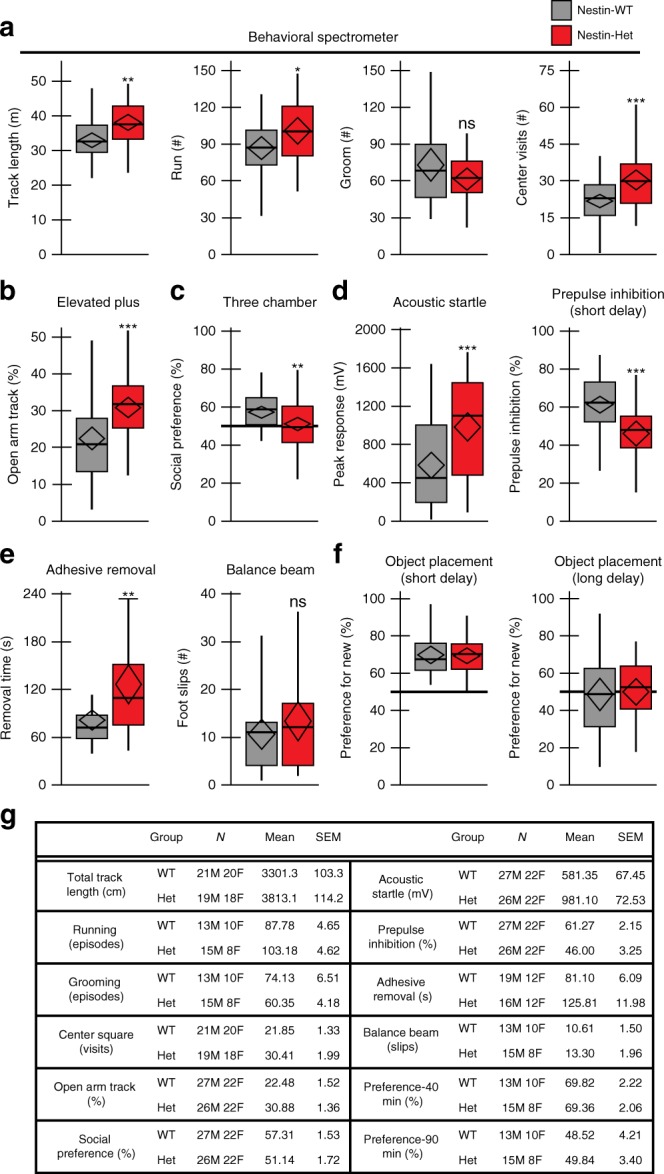
*Anks1b* heterozygous mice recapitulate phenotypes in *ANKS1B* haploinsufficiency syndrome. **a** In the Behavioral Spectrometer, Nestin-Het mice covered more track in 9 min and had more episodes of running than Nestin-WT controls. Reduction in grooming behavior was not statistically significant. Nestin-Het mice visit the center square of an open field more often than controls. **b** Nestin-Het mice showed a robust reduction in avoidance behaviors, covering more track in the open arms as a percentage of total track in open and closed arms of an elevated plus maze. **c** In the three-chamber test, Nestin-Het mice show significantly reduced and borderline preference for a conspecific mouse over an inanimate object. **d** Peak magnitude of the acoustic startle reflex is robustly increased in Nestin-Het mice. Sensorimotor gating measured by the percentage of prepulse inhibition (prepulse stimulus 40 ms before startle stimulus) is significantly reduced. **e** In a test of fine-motor dexterity, Nestin-Het mice require more time to remove adhesive from the forepaw. Nestin-Het mice do not exhibit significant deficits in gross motor coordination as measured by slips on a balance beam. **f** Nestin-Het mice do not show learning deficits in the object placement test, a hippocampus-dependent memory assay. Both groups averaged above a passing score (>50% preference for new location) using a 40-min retention interval. Raising the task difficulty by increasing the retention interval to 90 min did not result in a difference: mean values indicate that mice of both genotypes failed the test at 90 min (<50% preference). Reference line indicates 50% preference. Box plots show the mean and 95% confidence intervals (black diamond), median (black line), 25th–75th quantile (gray or red bar), and range (black whiskers). If two-way ANOVA showed significant main effect of genotype, post hoc two-sided Student’s *t*-test was performed, *p < 0.05, **p < 0.01, ***p < 0.001, ns = *p* > 0.05. **g** Numbers and sex of mice tested with the measures for each behavioral assay are shown as the mean and SEM. No sex-dependent effects of genotype were observed in any behavioral test by two-way ANOVA (Supplementary Data 5)

To test the effects of *Anks1b* deletion on an isogenic background, we performed a battery of behavioral assays on Nestin-Het mice across domains relevant to clinical features of *ANKS1B* haploinsufficiency syndrome (Fig. 3). Using the Behavioral Spectrometer, a system validated to assess aberrant activity patterns in mouse models of ASD^38^, we found that Nestin-Het mice covered greater distances and had increased episodes of running activity compared to Nestin-WT controls, consistent with a hyperactivity phenotype (Fig. 3a). No significant differences were observed in the number of grooming episodes, a correlate of stereotypies in ASD, or in orienting and rearing (Supplementary Fig. 3a). However, Nestin-Het mice showed an increase in number of visits, time, and percentage of total track in the center square of an open field, despite no difference in rearing (risk assessment), suggesting a reduction in anxiety. To test anxiety-related behaviors more robustly, we used the elevated plus maze, an assessment of unconditioned approach-avoidance conflict^39^. Nestin-Het mice spent increased time and distance in the open arms of the maze as a percentage of total track, confirming a decreased anxiety phenotype (Fig. 3b and Supplementary Fig. 3b). This effect was not generalizable to another measure of mood dysregulation, the Porsolt forced swim test of learned helplessness in depression (Supplementary Fig. 3c).

In the three-chamber test of social approach, an assay widely used to evaluate mouse models of autism^40^, Nestin-Het mice showed significantly reduced preference for a conspecific mouse over an inanimate object, and notably demonstrated borderline preference (51.1 ± 1.7%, mean ± SEM) for social interactions (Fig. 3c). Nestin-Het mice had a significantly higher likelihood of failing the social preference test as defined by spending less than 50% of total exploration time with the animal (Supplementary Fig. 3d). While Nestin-WT mice spent significantly more time exploring the animal than the object, Nestin-Het mice showed no difference between time spent sniffing the animal and time spent with the object (Supplementary Fig. 3d). While impaired sociability is the principal behavioral deficit observed in autism mouse models^40^, we performed additional testing for sensorimotor deficits, which often accompany an ASD diagnosis and are notable features of *ANKS1B* haploinsufficiency syndrome. In measures of the acoustic startle reflex, Nestin-Het mice showed a robust increase in the peak magnitude of the startle response (Fig. 3d). They also demonstrated marked impairments in prepulse inhibition (PPI) of the response, a measure of sensorimotor gating (Fig. 3d and Supplementary Fig. 3e). Deficits in startle and PPI have been described in several mouse models of autism^40^. Nestin-Het mice displayed significant deficits in fine-motor dexterity assayed by the adhesive removal test^41^ (Fig. 3e). Gross motor coordination was comparable to controls as measured by slips on a wooden balance beam, although we observed a trend toward increased slips in the Nestin-Het mice.

Despite our previous finding that AIDA-1 regulates synaptic plasticity in the hippocampus^25^, Nestin-Het mice showed no cognitive deficits. In the object placement test, a hippocampus-dependent learning task^39^, Nestin-Het mice showed no difference in preference for a novel object location compared to controls, a result consistent between retention intervals in which controls passed (short delay) or failed (long delay) (Fig. 3f). Statistical analyses for the 46-97 adult mice tested demonstrate robust results with high power for tests in which differences were significant. No sex differences were observed in any of the behaviors evaluated (Fig. 3g and Supplementary Data 5). To determine whether similar phenotypes could be observed earlier in development^42^, we tested Nestin-Het pups at pre-weaning ages. To test for the emergence of social, sensory, and motor deficits, we performed homing, negative geotaxis, and acoustic startle tests in pups from P10 to P16. No deficits or delays were observed for Nestin-Het mice in these developmental milestones compared to Nestin-WT and other littermate control groups (Supplementary Fig. 3f and Supplementary Data 5). To test for social communication deficits, we analyzed ultrasonic vocalizations (USVs) recorded from maternally separated pups at P8 and P10. No significant changes in frequency, syllable counts and intervals, or syllable repertoires were detected between Nestin-Het pups and three other littermate control groups (Supplementary Fig. 3g–i).

### AIDA-1 interactome reveals novel cellular roles for *ANKS1B*

**Fig. 4:**
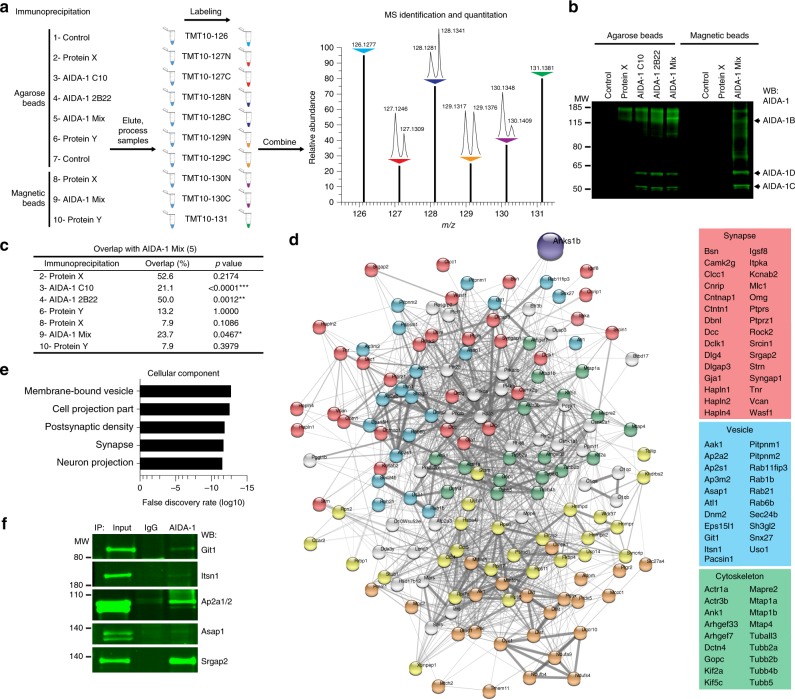
Identification of the AIDA-1 interactome by mass spectrometry reveals novel cellular roles. **a** Ten immunoprecipitations (IPs), including four IPs using different AIDA-1 antibodies (C-10, 2B22, or Mix) and beads (agarose or magnetic), two control IPs using mouse IgG, and four control IPs of unrelated synaptic proteins. Samples were differentially labeled using isobaric tags and mixed for MS analysis. Peptides bearing each isobaric tag were simultaneously identified and quantified. **b** Western blots showing AIDA-1 isoforms immunoprecipitated by AIDA-1 antibody combinations. Only antibody 1A11 (included in AIDA-1 Mix) was able to pull down AIDA-1B. **c** Overlap of interactors from the AIDA-1 Mix Agarose sample was significant only with interactors from other AIDA-1 IPs by two-sided Fisher’s exact test, *p < 0.05, **p < 0.01, ***p < 0.001. **d** Depiction of the AIDA-1 interactome prominently featuring components of the synaptic compartment, membrane-bound vesicles, and cytoskeletal projections. (Yellow = ribosome and proteasome; Orange = mitochondria). **e** Top gene ontology (GO) terms for cellular components enriched in the AIDA-1 interactome. **f** Co-IP of novel AIDA-1 interactors from mouse brain lysates. Git1 = 84 kDa, Itsn1 = 195 kDa, Ap2a1/2 = 108/104 kDa, Asap1 = 125 kDa, Srgap2 = 121 kDa

Our findings demonstrate that loss of AIDA-1 expression leads to neurobehavioral phenotypes in both human and mouse. To obtain insight into the molecular mechanisms that link AIDA-1 to disease, we identified the AIDA-1 interactome in mouse brain using quantitative proteomic methods (Fig. 4a). We performed ten unique immunoprecipitations (IPs) from mouse brain lysates using control IgGs, AIDA-1 IgGs, and antibodies to unrelated synaptic proteins crosslinked to protein G-agarose or magnetic beads. Each eluted IP sample was labeled with a unique isobaric tag within a 10-plex set of tandem mass tags^43^. This approach allowed us to accurately quantify identified peptides, simultaneously analyze all samples to reduce variability associated with mass spectrometry (MS) analysis, and rigorously control for nonspecific binding by normalizing to control IgG samples and comparing to unrelated protein interactomes (Supplementary Data 6a). We used a commercially available antibody (C-10, Santa Cruz Biotechnologies), as well as in-house antibodies known to immunoprecipitate AIDA-1 (1A11 and 2B22) (Fig. 4b). We defined the AIDA-1 interactome as proteins co-immunoprecipitated by AIDA-1 antibodies that were identified and quantified by ≥3 peptides and enriched >1.4-fold over background in agarose experiments, or >3.0-fold in magnetic bead experiments (Supplementary Data 6b). Determination of these cutoff values is explained in Methods. To gauge the specificity of the AIDA-1 interactome, we analyzed the overlap between AIDA-1 and unrelated interactomes using two-sided Fisher’s exact test. Since co-IP for synaptic proteins X and Y are likely to yield similar protein populations, we used a stringent statistical background for the hypergeometric distribution limited to proteins enriched in any co-IP sample (255 total proteins). As expected, the interactors obtained using a mixture of AIDA-1 antibodies (AIDA-1 Mix Agarose) showed significant overlap only with those from other AIDA-1 samples (Fig. 4c).

We analyzed the AIDA-1 interactome using StringDB^44^ for a general overview of gene ontology (GO) enrichments (Fig. 4d and Supplementary Data 7). The interactome contained proteins from cellular components where AIDA-1 is known to localize, including the synaptic and postsynaptic compartments of neuronal projections. However, the most significant enrichment was in components of membrane-bound vesicles (Fig. 4e). In line with this localization, our previous work demonstrated impaired NMDAR subunit trafficking through the endoplasmic reticulum (ER) in *Anks1b* conditional knockout mice^25^. Although the mechanism of this regulation is unknown, we had previously shown that AIDA-1 is associated with intracellular vesicles in the presynaptic and postsynaptic compartments^20^. To confirm the vesicular distribution of *ANKS1B*-encoded proteins, we performed full subcellular fractionation of postmortem human brain and mouse brain tissue. Western blots show that AIDA-1 isoforms are enriched in microsomes derived from light membrane fractions, as well as PSD fractions (Supplementary Fig. 4). Characterization of these microsomal fractions shows that they are enriched in the endoplasmic reticulum marker calnexin^45^ as well as small GTPases (Rab11) that regulate trafficking through recycling endosomes^46^. To validate novel AIDA-1 interactors, we performed independent co-IP experiments in mouse brain lysates and confirmed the association of AIDA-1 with Git1^47^, Itsn1 and Ap2a1/2^48^, Asap1^49^, and Srgap2^50^, proteins that regulate membrane trafficking and synaptic development (Fig. 4f).

### AIDA-1 interactome links *ANKS1B* to development and disease

**Fig. 5:**
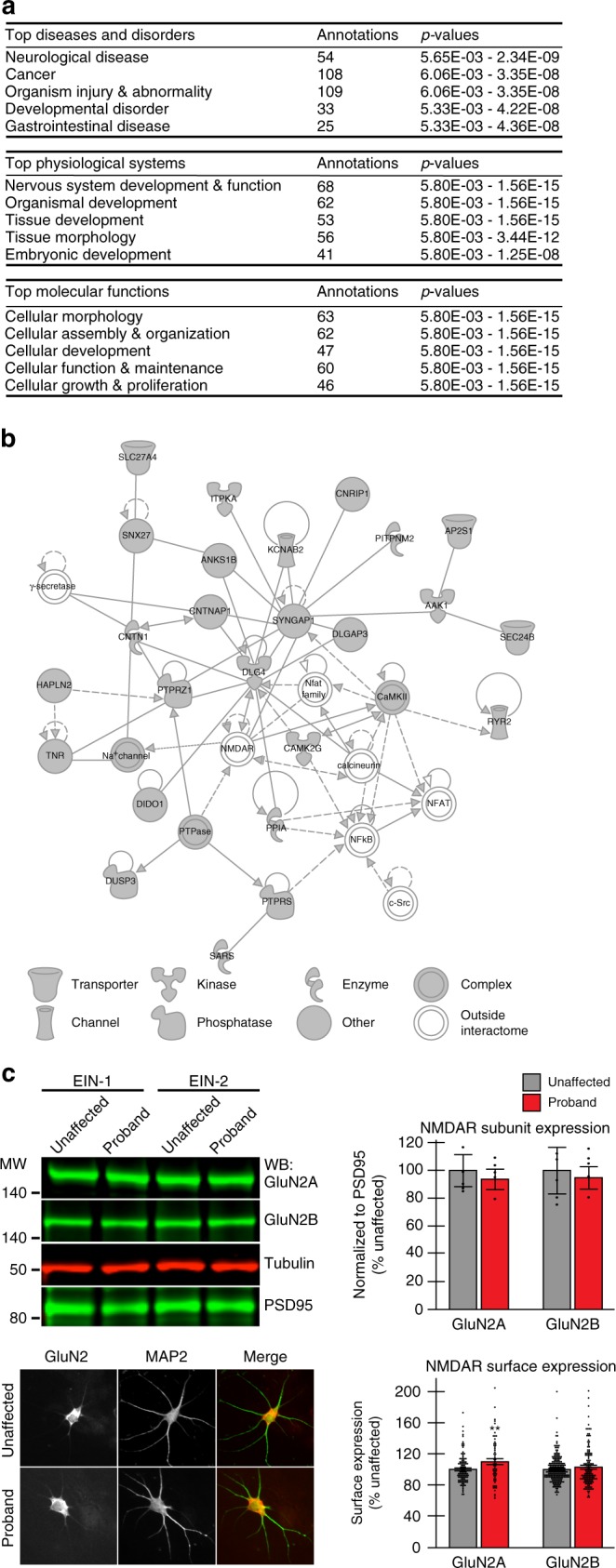
Analysis of the AIDA-1 interactome yields functional pathways and mechanisms of disease. **a** Hierarchical analysis of the most significant diseases and functions in IPA reveals the top disorders, physiological systems, and cellular processes regulated by the AIDA-1 interactome (*p*-values are given as a range for the diseases and functions annotated in each category). **b** The top network identified using Ingenuity Pathway Analysis (IPA) revealed known interactors and novel pathways associated with AIDA-1 (network score = 49, number of focus molecules = 25). Solid lines = direct interaction, dashed lines = indirect interaction, filled arrows = activation, open arrows = translocation, dash = inhibition. **c** (Top) Western blot (Family EIN-1 and EIN-2) and quantitation (Family EIN-2) of NMDAR subunits GluN2A and GluN2B in iPSC-derived neurons show no changes in probands (10 μg lysate). *N* = 3 biological replicates. (Bottom) Sample images (GluN2B) and quantitation of GluN2A and GluN2B surface expression in neurons from proband EIN-2-1 and unaffected mother EIN-2-M reveal a significant increase in GluN2A, but no change in GluN2B. *N* = 3 biological replicates based on 60–99 neurons. Scale bar = 10 μm. Bar graphs show mean ± SEM, two-sided Student’s *t*-test, *p < 0.05, **p < 0.01

We identified the most significantly regulated diseases and functions in the AIDA-1 interactome (Supplementary Data 8a), then performed higher-order analyses to categorize these annotations using Ingenuity Pathway Analysis (IPA, QIAGEN Bioinformatics) (Fig. 5a). Consistent with clinical findings in *ANKS1B* haploinsufficiency syndrome, Neurological Disease and Developmental Disorder were among the top hits in the category of Diseases and Disorders (Supplementary Data 8b). In the category of Physiological Systems, the most significant effects were found on development, including nervous system development and function. Top molecular functions of the interactome converged on cellular processes, including cell morphology and cellular development. Advanced pathway construction in IPA identified a functional network with predicted functions in cell-to-cell signaling and interaction, cell morphology, and nervous system development and function as the top-scoring network (Fig. 5b and Supplementary Data 8c). Inspection of individual proteins in this network revealed known AIDA-1 interactors, including PSD95^23^, SynGAP1^51^, and SNX27^52^. These and other molecules converge on the NMDA receptor as a major node in the pathway. Based on this network analysis, the interaction of AIDA-1 with proteins that regulate vesicle transport (Fig. 4e, f and Supplementary Data 4), and our work linking AIDA-1 to NMDAR function^25^, we measured surface expression of the NMDAR subunits GluN2B and GluN2A in iPSC-derived neurons using immunofluorescence. Contrary to our findings in forebrain *Anks1b* knockout mice^25^, we found no reduction in surface GluN2B levels in proband neurons. However, we found that GluN2A subunits showed a significant increase in surface expression, which is consistent with our previous findings (Fig. 5c). No changes in overall GluN2B and GluN2A levels were detected by western blot, suggesting that *ANKS1B* haploinsufficiency leads to aberrant trafficking of NMDAR subunits in patient-derived neurons. These results indicate that altered NMDAR localization is a possible mechanism of disease in *ANKS1B* haploinsufficiency syndrome. From the identity of individual molecules to their predicted roles in disease, our analysis of the AIDA-1 interactome predicts participation of *ANKS1B* in cellular processes crucial to normal development and implicated in the etiology of neurodevelopmental disorders.

## Discussion

In this study, we discovered that microdeletions in the *ANKS1B* gene encoding AIDA-1 lead to a neurodevelopmental syndrome characterized by autism, ADHD, and speech and motor impairment. Ours is the first to report patients with monogenic deletions in *ANKS1B* and establish the contributions of these rare CNVs to disease. None of the variants we report have been identified in control populations^30^. However, most *ANKS1B* deletions were inherited from parents reported to have normal or mild phenotypes (Table 1 and Supplementary Data 2). Variable penetrance is a common feature of CNVs in neurodevelopmental disorders and could be explained by inherited risk factors unmasked by deletions, or by compensation strategies employed throughout development^3,4^. Through comprehensive clinical interviews and testing, we found previously undiagnosed neurodevelopmental phenotypes in affected fathers from families EIN-1 and EIN-2. In both cases, fathers showed impairments in similar domains as their affected children and other probands, demonstrating that the phenotype segregated with the *ANKS1B* deletion. The degree to which the deletion impaired adaptive function was related to developmental age: parents rated younger affected children as having more difficulty with daily function compared to older children. Our results highlight the need for deep clinical phenotyping in addition to genomic sequencing to identify links between CNVs and phenotypes, especially in spectrum disorders with variable presentations and developmental compensation. Despite the range of phenotypes and the rarity of *ANKS1B* mutations, we identified 12 families with shared features, supporting a causal link between *ANKS1B* deletion and the clinical syndrome. We also identified *ANKS1B* deletions in patients with CNVs overlapping other genes. Similarities between the neurodevelopmental disorders in these individuals and those with monogenic *ANKS1B* deletions suggests that *ANKS1B* haploinsufficiency contributes to clinical presentation in these patients.

*ANKS1B* encodes the protein AIDA-1, which is highly enriched in the brain and specifically expressed in neurons. We induced differentiation of iPSCs into excitatory neurons to enable further investigation of synaptic dysfunction and aberrant neuronal development, which are proposed mechanisms for neurodevelopmental disorders^53^. Using neurons from different families, we correlated microdeletions overlapping distinct *ANKS1B* exons with similar reductions in encoded AIDA-1 protein (Fig. 1f and Supplementary Data 4). Expression of all major AIDA-1 isoforms and *ANKS1B* transcripts are reduced, including those with putative transcription start sites downstream of microdeletions. Reduced AIDA-1D and AIDA-1C protein isoforms may reflect the loss of unidentified *cis*-acting regulatory elements in the deleted N-terminal regions of the *ANKS1B* gene. Alternatively, AIDA-1 isoforms may stabilize each other, such that loss of the larger protein leads to the degradation of smaller isoforms. Additional work will be needed to elucidate this potential isoform-dependent regulation of *ANKS1B* expression.

Patient-derived iNs and Nestin-Het mice both demonstrate reduction in all major isoforms and transcripts (Fig. 1g, h and Supplementary Fig. 2c, d). This suggests broad dysfunction in AIDA-1-regulated processes across brain regions: *ANKS1B* transcripts are differentially spliced in Wernicke’s area, important for language development, and the prefrontal cortex, implicated in neurodevelopmental disorders including autism^54^. AIDA-1 is strongly expressed in the cerebellum^20^, especially the large isoform AIDA-1B (Fig. 2b). *Anks1b* transcripts are present in Purkinje cells of the developing cerebellum^55^, consistent with the striking motor phenotypes in patients and the emerging role of the cerebellum in language and social development^56^. Although gross characterization of cerebellar size revealed no significant changes in *Anks1b* Nestin-Het mice (Supplementary Fig. 2e), our gene expression and behavioral data demonstrate that *ANKS1B* deletions could affect development and function in brain regions crucial for motor coordination and social communication.

Heterozygous *Anks1b* deletion in mice produced impaired social interactions, altered activity patterns, and sensorimotor deficits reminiscent of *ANKS1B* haploinsufficiency syndrome. We did not observe the impairment in hippocampus-dependent spatial memory that would be expected after loss of a gene required for hippocampal synaptic plasticity^25^. Heterozygous knockdown of *Anks1b* may not have been sufficient to produce changes in plasticity, since our previous work used a homozygous forebrain knockout model. Moreover, intellectual disability is an inconsistent feature in patients. Motor deficits in speech production and fine-motor control are prominent features of the syndrome, reflected in reduced dexterity in Nestin-Het mice. Gross motor coordination was not significantly impaired in the balance beam test, although a trend was observed toward increased slips. Impaired sociability, altered approach-avoidance behaviors, hyperactivity, increased startle sensitivity, and reduced prepulse inhibition have all been described in mouse models of autism, including *Fmr1* knockout and the BTBR strain^40^. Our findings of neurobehavioral abnormalities are largely consistent with a previous evaluation of ketamine sensitivity in a homozygous *Anks1b* deletion model^57^. Notably, we found that heterozygous deletion with ~50% residual AIDA-1 expression is sufficient to observe these behavioral effects, supporting an *ANKS1B* haploinsufficiency model in the etiology of the clinical syndrome. In contrast to *ANKS1B* deletion probands, *Anks1b* Nestin-Het mice did not demonstrate social, sensory, or motor impairments in early development (Supplementary Fig. 3f–i). However, developmental stages in mice correlate imperfectly with the timing of human development, and an initial period of normal development has been described in other mouse models of syndromic neurodevelopmental disorders^58^. Since the Nestin-Het model essentially reproduces a heterozygous *Anks1b* microdeletion on an isogenic background, our results strengthen the causal link between *ANKS1B* deletion and neurodevelopmental disorders in probands.

Through co-immunoprecipitation and quantitative mass spectrometry, we defined the AIDA-1 interactome with significant reproducibility. The novel methodology we present here, involving simultaneous analysis of multiple internal controls, increases our confidence in the AIDA-1 interactors identified. An unbiased approach using whole brain homogenate enabled us to identify novel interactors and striking enrichment in components of membrane-bound vesicles, including Rab family regulators of recycling and contents of the AP-2 endocytic complex. Fractionation and co-immunoprecipitation with key regulators of vesicle transport validated this approach. Higher-order analysis of the AIDA-1 interactome in IPA traced converging functions of *ANKS1B* in normal development and disease-related processes (Supplementary Data 8b). Comparing our proteomic data to transcriptomic and genomic studies identifying *ANKS1B* as a gene of interest leads to several intriguing parallels. In diseases and disorders, neurological disease is the top category: aside from the neurodevelopmental and neuropsychiatric disorders in *ANKS1B* haploinsufficiency syndrome, literature also suggests a role in dementia^59^ and epilepsy^60^. In physiological systems, major roles for the AIDA-1 interactome in organismal and embryonic development is consistent with *ANKS1B* influence on body size^61^, gestational development^62^, and our current findings of reduced body mass and perinatal lethality in *Anks1b* knockout mice. For nervous system development and function, the annotation of highest significance is neuritogenesis (IPA, one-sided Fisher’s exact test, *p* = 1.56e-15), which is represented in all top molecular functions of the AIDA-1 interactome. Finally, top canonical pathways regulated by the AIDA-1 interactome include axon guidance signaling and endocytic pathways, which are supported by our co-IP and fractionation results. These results place *ANKS1B* among genes that link endosomal trafficking to neurodevelopmental disorders^63,64^.

Analysis of the top network in the AIDA-1 interactome reconciles known and novel roles for AIDA-1: postsynaptic scaffolds (PSD95, DLGAP3), vesicle components (Snx27, AP-2), enzymatic switches (SynGAP1, AAK1), and membrane proteins (NMDAR, Contactin-1) are all major players. The pathway features prominent regulators of synaptic function implicated in neurodevelopmental disorders, including NMDARs (autism)^65^, SynGAP1 (autism and ID)^66^, and Snx27 (Down Syndrome)^67^. Given that AIDA-1 participates in the localization of GluN2A and GluN2B^25^, we tested the hypothesis that changes in NMDAR expression underlie *ANKS1B* haploinsufficiency syndrome. Although surface GluN2A was significantly increased in proband-derived neurons, we did not observe changes in GluN2B. This discrepancy may reflect dose-dependent or synapse-specific effects: in contrast to homozygous AIDA-1 forebrain knockout mice, all human microdeletions are heterozygous, proband neurons retain ~50% of AIDA-1, and total surface expression was measured (Fig. 1g). Moreover, GluN2B is the primary subunit expressed in iPSC-derived neurons^68^: regulatory mechanisms maintaining GluN2B expression could compensate for partial loss of AIDA-1. Divergent mechanisms may regulate NMDAR subunit composition in humans and mice, highlighting the importance of developing this human-based model^69^ of *ANKS1B* haploinsufficiency syndrome. Overall, we showed that *ANKS1B* haploinsufficiency leads to changes in NMDAR subunit localization, validating the functions predicted by our previous work and our current analysis of the AIDA-1 interactome.

We identified *ANKS1B* as a crucial gene for human development in which rare loss-of-function variants cause neurodevelopmental disorders, including autism, ADHD, and deficits in speech and motor function. Through proteomic analysis, we defined a network of molecular pathways through which AIDA-1 interactors regulate neuronal function and identified a specific deficit in NMDAR subunit localization as a possible mechanism of disease. By generating neurons from patients with *ANKS1B* haploinsufficiency syndrome and the *Anks1b* Nestin-Het mouse model, we have developed tools to further probe the mechanisms by which AIDA-1 enables normal brain development and *ANKS1B* haploinsufficiency contributes to neurodevelopmental disorders.

## Methods

### Human subjects and clinical phenotyping

Clinical phenotyping received ethical approval by the Institutional Review Board (IRB) at Albert Einstein College of Medicine in IRB protocol #2011-320 to S.M. Informed consent was obtained from all participants. Patients were identified through direct contact (EIN-1 and EIN-2), the Autism Speaks MSSNG project (TOR-1 and TOR-2), the DECIPHER database (DEC-1 through DEC-12), and clinicians contacted by the GeneMatcher online resource (GEN-1). Families EIN-1 and EIN-2 were enrolled in a protocol approved by the Institutional Review Board at Albert Einstein College of Medicine. Clinical interviews and exams, neuropsychological tests, and family questionnaires were administered at the Albert Einstein College of Medicine Human Clinical Phenotyping Core by licensed neuropsychologists. Evaluation of children was conducted in the morning, while evaluation of adults was conducted from morning until afternoon. All subjects were given a lunch break at noon, and additional breaks were provided when requested. Subjects completed a neuropsychological battery consisting of tests appropriate to developmental age. Tests generated a standard score (mean = 100, standard deviation = 15), *T*-score (mean = 50, standard deviation = 10), scaled score (mean = 10, standard deviation = 3), *z-*score (mean = 0, standard deviation = 1), or percentile normed for age. To test cognitive function, the Wechsler Primary Preschool Scale of Intelligence, Fourth Edition (WPPSI-IV); Wechsler Scale of Intelligence for Children, Fifth Edition (WISC-V); or Wechsler Adult Intelligence Scale, Fourth Edition (WAIS-IV) was used. Verbal reasoning was assessed by the Verbal Comprehension Index (VCI) of the Wechsler system. Depending on age, perceptual reasoning and visual spatial reasoning skills were either subsumed by a single Perceptual Reasoning Index (PRI) or separated into the Visual Spatial Index (VSI, the ability to synthesize visual information) and Fluid Reasoning Index (FRI, the ability to identify a solution based on patterns). Working Memory Index (WMI) and Processing Speed Index (PSI) were measured in the Wechsler system if age-appropriate. From the Wechsler Individual Achievement Test, Third Edition (WIAT-III), the Word Reading, Spelling, and Numerical Operations subtests were given to assess academic level.

Speech and language abilities were assessed using the Boston Naming Test (BNT), as well as subscales of the Developmental NEuroPSYchological Assessment, Second Edition (NEPSY-II), and Clinical Evaluation of Language Fundamentals, Fifth Edition (CELF-V). Motor function was assessed by the Purdue Pegboard Test and the Movement Assessment Battery for Children (Movement ABC). For adult subjects, the Movement ABC was administered and scored for the oldest normed group (16-years-old). To discriminate between perceptual and fine-motor problems, the Beery Buktenika Developmental Test of Visual-Motor Integration, Sixth Edition (VMI-VI) was given. To assess memory, the Rey Complex Figure Test (RCFT) was given for visuospatial organization and visual memory, and the California Verbal Learning Test, Second Edition (CVLT-II) or California Verbal Learning Test, Children’s Version (CVLT-C) was given for verbal memory. Processing speed was assessed using the oral response task on the Symbol Digit Modalities Test (SDMT) to reduce the effects of motor dysfunction. Conners’ Continuous Performance Test, Third Edition (CPT-III) and Conners’ Kiddie Continuous Performance Test, Second Edition (K-CPT-II) were given to assess sustained attention. Select subtests from the Delis-Kaplan Executive Function System (D-KEFS) and NEPSY-II were given to assess executive function and social communication, respectively. The Autism Diagnostic Observation Schedule, Second Edition (ADOS-II) was administered to all subjects. To assess sensory, emotional, social, and adaptive function in children, parents completed the Sensory Profile, Second Edition; Behavior Assessment System for Children, Second Edition (BASC-II); Social Responsiveness Scale, Second Edition (SRS-II); and Vineland Adaptive Behavior Scales, Third Edition (Vineland-III).

### Whole- exome sequencing

Genomic DNA was isolated from whole-blood samples using the QIAamp DNA Blood Mini Kit (Qiagen) with RNAse A. Each sample was analyzed using NimbleGen SeqCap EZ Exome Library v3.0 64 Mb (300 bp average insert size) to capture the exome. Paired end sequencing was completed using Illumina platforms. Before mapping reads to GRCh37/hg19 (Genome Reference Consortium), fastq data reads were trimmed of sequencing adapters and filtered for sequence quality using trimGalore against the standard Trueseq adaptor. Reads were aligned to GRCh37/hg19 using BWA, then SAMtools commands were used to clean up paired read information and sort bam files. SAMtools fixmate was used to ensure consistent information display for both reads in a pair, then SAMtools sort to order the output file according to genomic sort order. After mapping, the Picard MarkDuplicates command was used to remove or mark PCR duplicates. BEDtools intersect was used to calculate the number of reads, which overlap a target BED file by at least 1 bp, yielding an output of the number of on-target reads. Dividing this number by the total number of mapped, non-duplicate reads yielded the percentage of on-target reads. The GATK function DepthOfCoverage was used to calculate depth of coverage metrics over enrichment targets, including mean, median, and percent of target bases covered at a depth of at least 30 × . To calculate read depth of *ANKS1B* exons in probands, exons within the *ANKS1B* gene were taken as enrichment targets, the counts were gathered for each exon group (1–9, 10–14, and 15–26), and read depths were normalized to the unaffected mother in each trio. To generate the lists of segregated loss-of-function single-nucleotide variants (SNVs) in affected individuals, the list of SNVs for each sample was merged with the ClinVar database of disease annotations. SNVs are listed in Supplementary Data 3 after filtering for High predicted impact on gene expression, then for annotation with specified ClinVar phenotype(s), and finally for segregation with the *ANKS1B* deletion (present in proband and affected father but not in affected mother).

### Tissue samples and antibodies

Hippocampal sections from control postmortem human tissue were obtained from the University of Maryland repositories of the NIH NeuroBioBank. Antibodies used for western blotting were AIDA-1 C-10 (1:1000, Santa Cruz), tubulin (1:1000, Thermo Fisher), PSD95 (1:1000, NeuroMab), GAPDH (1:1000 Cell Signaling Tech), calnexin (1:1000, Genscript), Rab11 (1:1000, Cell Signaling Tech), Git1 (1:1000, Santa Cruz), Itsn1 (1:1000, Santa Cruz), AP2a1/2 (1:000, Santa Cruz), Asap1 (1:1000, Santa Cruz), and Srgap2 (1:1000, Proteintech). Antibodies for immunocytochemistry were Oct-4 (1:1000, Abcam), Sox-2 (1:500, Cell Signaling Tech), MAP-2 (1:1000, EnCor), GluN2B (1:500, Alomone), and GluN2A (1:500, Alomone). Mouse monoclonal antibodies 1A11 and 2B22 were developed and validated in house^25^. Chicken AIDA-1 antibody 5707 was generated by Aves Labs to the peptide sequence GDRLHDDPPQKPPRC at the end of the second SAM domain of AIDA-1.

### Human-induced pluripotent stem cells and induced neurons

IPSC generation received ethical approval by the Institutional Review Board (IRB) at Albert Einstein College of Medicine in IRB protocol #2017-8311 to B.A.J. Informed consent was obtained from all blood donors. Peripheral blood samples were collected from families EIN-1 and EIN-2, and peripheral blood mononuclear CD34 + cells (PBMCs) were used to generate human-induced pluripotent stem cells^33^ (iPSCs) at the Albert Einstein College of Medicine Stem Cell Core. Three iPSC clones from each individual were generated from CD34 + cells using CytoTune-iPS 2.0 Sendai Reprogramming Kit (Invitrogen) following the manufacturer’s protocol. Briefly, frozen PBMCs were thawed 2 days before reprogramming (Day -2) and cultured in STIF medium. On Day 0, CD34 + cells were flow-sorted by FACSAria II (BD) and transduced with Sendai virus vector of KOS (*hKlf4*, *hOct3/4*, and *hSox2*) at multiplicity of infection (MOI) 5, 5, and 3, respectively, in the presence of Polybrene 4 μg/mL. Three days after transduction, transduced cells were plated on Matrigel-coated 24-well plates in StemSpan SFEM medium (STEMCELL Technologies). Essential 8 (E8) medium (Invitrogen/Thermo Fisher) replaced half of StemSpan SFEM medium on Day 7 and replaced it completely on Day 8. Culture medium was changed every 2 days from Day 2 to Day 7 and changed daily from Day 8. The iPS-like clones were picked and passaged by mechanical dissection from Day 21 to Day 28. FACS analysis of pluripotency markers SSEA3, SSEA4, TRA-1-60, and TRA-1-81; in vitro differentiation and immunohistochemical detection of germ-layer markers α-fetoprotein, α-smooth muscle actin, and β-III tubulin; RT-PCR assay for virus gene integration; and karyotyping were performed on each iPSC clone to ensure the quality of integration-free iPSCs. Clones were maintained in E8 as described by the manufacturer with occasional removal of differentiated cells. IPSC identity was periodically confirmed by immunocytochemistry using pluripotency markers Oct-4 and Sox-2.

Induced neurons (iNs) were generated by forced expression of the human neuronal transcription factor neurogenin-2 (*NGN2*) in iPSCs and growing cells in the presence of neurotrophic factors and rat astrocytes to facilitate maturation into excitatory neurons^34^. Briefly, iPSCs were treated with trypLE, dissociated for 5 min, and plated on Matrigel-coated plates in E8 medium containing ROCK inhibitor 1 µM (H-1152 dihydrocholoride, Santa Cruz). Cells were plated on Matrigel-coated 24-well plates for western blotting, and on Matrigel-coated coverslips for immunocytochemistry. On Day 2, cells were infected with lentivirus (*NGN2* and rtTA, Addgene) in E8. On Day 3, media was replaced with DMEM/F12 (Thermo Fisher) containing N-2 1% (Invitrogen), NEAA 1% (Thermo Fisher), brain-derived neurotrophic factor (BDNF) 10 ng/mL (GerminiBio), NT-3 10 ng/mL (GerminiBio), laminin 0.2 µg/mL (Santa Cruz), and doxycycline 4 µg/mL (Santa Cruz). On Day 4, cells were selected in puromycin 1 μg/mL for 24 h. On Day 5, rat astrocytes were added in DMEM/F12 containing BDNF, NT-3, and 0.5 µM Ara-C (Santa Cruz). On Day 6, medium was replaced with Neurobasal medium (Thermo Fisher) supplemented with B-27 (Thermo Fisher), GlutaMAX (Thermo Fisher), BDNF, NT-3, and doxycycline. On Day 10, 2.5% fetal bovine serum was added to the medium. On Day 19, iNs were lysed or fixed for analysis.

### Imaging

For histological analysis, adult mice at 16 weeks of age were perfused with 4% PFA. Brains were collected and medial sagittal brain sections (40 µm thick) were prepared and processed for cresyl violet-based Nissl staining. Images were acquired on a Zeiss Axio Scan.Z1 slide scanner with a 5x objective. Areas were quantified using ZEN software (blue edition). Immunofluorescence of iPSC-derived neurons was performed using standard procedures and imaged on a Zeiss AxioObserver microscope at 20x. To measure surface NMDAR subunits, Day 28 induced neurons were fixed and immunostained with GluN2A or GluN2B antibodies, then permeabilized with 0.01% Triton X-100 and immunostained with the neuronal and dendritic marker MAP2. All measurements were obtained and quantified by an experimenter blind to genotype.

### Reverse transcription quantitative polymerase chain reaction (RT-qPCR)

RNA was isolated from iPSC-derived neuronal cultures and from Nestin-Het and Nestin-WT mice using the RNeasy Plus Mini Kit (Qiagen). Primers were designed to *ANKS1B* transcripts for *Exon 4* (forward: 5′-GCCCTACACTGTGCAGCTCAATA-3′, reverse: 5′-GGGTCAGTGAGCTCTTCTAGGAG-3′), *Exon 13* (forward: 5′-ACCATACCATTGTTGGCACAAG-3′, reverse: 5′-ACAAATCCCCCTGCGTTCAT-3′), and *Exon 20* (forward: 5′-ACCTCCGAATGAAGCCACAG-3′, reverse: 5′-GCTTTGTAATCACACGACTGGA-3′). In all experiments, the neuronal marker *MAP2* (forward: 5′-TTGGTGCCGAGTGAGAAGA-3′, reverse: 5′-GTCTGGCAGTGGTTGGTTAA-3′)^70^ was used as the housekeeping gene.

### *Anks1b* Nestin-Het mouse model

To generate the *Anks1b* heterozygous conditional knockout line, male mice from the transgenic B6.Cg-Tg(*Nes-cre*)1Kln/J line (*Nestin-Cre*) were purchased from Jackson Laboratories (#003771) and crossed to female mice from the *Anks1b*^*flox*/*flox*^ line previously generated^25^. The Nestin-Het mouse line was made congenic by backcrossing to C57BL/6J mice (Jackson Laboratories, #000664) for at least ten generations prior to behavioral testing. For all western blotting, RT-qPCR, gross and histological morphometry, and behavioral assays, the *Nestin-Cre;Anks1b*^*wt/flox*^ mice generated from this colony were used as mutants (Nestin-Het) while *Nestin-Cre;Anks1b*^*wt/wt*^ mice (Nestin-WT) were used as controls.

### Animal behavioral assays

All experiments complied with all relevant ethical regulations for animal testing and research, and were approved by the Albert Einstein College of Medicine Institutional Animal Care and Use Committee (IACUC). Mice were housed and handled at the Albert Einstein College of Medicine and behavioral phenotyping was performed as five independent cohorts in the Albert Einstein College of Medicine Animal Behavior Core under the supervision of Dr. Maria Gulinello. In the Behavioral Spectrometer^38^, mice were recorded for 9 min in an open field with a center area of 18.0 × 18.0 cm. Elevated plus maze (5 min), forced swim test (9 min in 25 °C water bath), and three-chamber test (5 min with ovariectomized C57BL/6J mouse) were performed using standard procedures^71^. Social preference was calculated using the following formula: *social preference* *=* (*social sniffing time*)*/*(*social sniffing time + object sniffing time*) × *100%*. Acoustic startle reflex and prepulse inhibition of the startle response were assayed^72^ in a single session for each mouse using randomized, interleaved trials (five each) for acoustic startle response (115 dB) and prepulse inhibition (PPI). Short-delay PPI trials were conducted with an 81-dB prepulse 40 ms before the 115-dB target stimulus, while long-delay PPI was tested with a 200-ms inter-stimulus interval. Adhesive removal tests were performed^41^ as a single trial by applying a 0.5 × 4.0 cm strip of Micropore (3M) medical adhesive to the left forepaw after a 30-min habituation to an empty mouse cage. Balance beam assay for motor coordination was performed^73^ on a 1.2 cm round wooden beam after pre-training. The novel object placement test was performed^74^ using a 5-min training phase, 4-min testing phase, and short (40 min) or long (90 min) retention intervals. During the testing phase, preference for the new placement was calculated using the following formula: *preference for new = (time exploring new placement)/(time exploring new placement + time exploring old placement) × 100%*.

All behavioral testing in adult mice aged 3–4 months and in pups aged P8-P16 was performed with experimenter blind to genotype. Feeding, mixed-genotype group housing, light-dark cycles, and time of testing were controlled across all five cohorts^75^. Sample sizes were estimated in JMP (version 14, SAS) to be of sufficient power to show effects independently in either sex if main effect of genotype-sex interaction (*α* = 0.05) were detected in two-way ANOVA (Supplementary Data 5). For all behavioral tests, post hoc two-sided Student’s *t*-test was performed for tests in which genotype was a significant main effect in two-way ANOVA. For the three-chamber test, likelihood ratio tests were additionally performed for effects of Genotype and Sex on passing (>50% social preference) or failing (<50% social preference). Post hoc contingency testing included chi-squared analyses and two-sided Fisher’s exact test. To assess differences between social and object sniffing times, repeated-measures ANOVA was also performed using a within-subjects design with Social time and Object time as responses and Genotype and Sex as factors. Sniffing times were separated by Social/Object and genotype, and post hoc testing was conducted using Tukey Kramer Honest Significant Difference (HSD) test for multiple comparisons. Since no main effect of sex or genotype-sex interaction was observed in any behavioral test, males and females of each genotype were combined for all post hoc testing, and samples of *N* > 20 were used to satisfy the Central Limit Theorem in lieu of testing for normality^75^. Power analysis and least significant number (LSN) were calculated in JMP for post hoc two-sided Student’s *t-*tests on significant effects from two-way ANOVA analyses. Developmental milestone testing was conducted^42^ at P10, P12, P14, and P16: tests included homing time to nesting material in the home cage, negative geotaxis righting time at 45°, and acoustic startle reflex scoring (0 = none, 1 = ear twitch, 2 = head twitch, 3 = full body startle). ANOVA was performed to detect main effects and interactions of age, genotype, and sex: since age was an expected main effect and did not interact with any other factors, no post hoc testing was conducted. Ultrasonic vocalizations from maternally isolated pups were recorded at P8 and P10 using Avisoft Bioacoustics software for 6 min and analyzed using MUPET, an unsupervised machine-learning-based algorithm that analyzes vocalization parameters, classifies syllables into repertoires, and compares between test groups to generate a similarity score^76^.

### Multiplexed large-scale immunoprecipitations

To perform immunoprecipitations (IPs), we incubated 300 µg of antibodies with 200 µl of agarose (Protein G Sepharose) beads for 1 h at 4 °C in PBS, washed 2x with 0.2 M triethanolamine pH 8.2 (TEA), and then crosslinked using freshly made 30 mM dimethyl pimelimidate (DMP) in TEA for 25 min at room temperature (RT). Beads were washed for 5 min with TEA, crosslinked again using a fresh DMP solution, washed 3x with TEA, and quenched using two 10-min washes of 100 mM ethanolamine. Unbound antibodies were stripped off beads 3x using 100 mM glycine pH 3.1 for 5 min, and beads were then washed 2x with PBS and stored in PBS/NaN_3_. For crosslinking antibodies to magnetic beads, we coupled 90 µg of antibodies to 6 mg of Epoxy Dynabeads (Dynal) according to the manufacturer instructions. Antibodies used were: mouse IgG, AIDA-1 C-10 (Santa Cruz), AIDA-1 2B22, or AIDA-1 Mix (C-10 + 1A11 + 2B22). Each individual IP was split into two reactions: one IP consisted of 40 µg coupled antibodies incubated with 5 mg of total mouse brains (whole brain, including olfactory bulb, cortices, midbrain, and cerebellum dissected from 3-month-old C57BL/6J mice) lysed in a gentle dodecyl-β-maltoside buffer (DBM: 10 mM HEPES pH 7.4, 190 mM NaCl, 10 mM KCl, 1 mM EGTA, 1% dodecyl-β-maltoside), and the second IP consisted of 40 µg incubated with 5 mg of lysate in a harsher RIPA buffer (25 mM Tris pH 7.4, 300 mM NaCl, 1% Triton X-100, 0.5% deoxycholate, 0.1% SDS, 2 mM EDTA). This approach allowed for the capture of detergent-sensitive interactions (DBM) and additional interactions in detergent-resistant synaptic and nuclear structures (RIPA). We incubated IPs for 2 h at RT with rocking, added two volumes of either 10 mM HEPES (DBM lysates) or 25 mM Tris (RIPA lysates) to dilute salts and detergents, and incubated overnight at 4 °C. Beads were washed 5x with TBS/Tween-20, DBM and RIPA IPs for each antibody sample were mixed together, and bound proteins were eluted in 2x non-reducing Laemmli sample buffer at 70 °C for 30 min.

### Tandem mass tag (TMT) labeling of peptides

Ten micrograms of each immunoprecipitation sample was electrophoresed briefly (dye front 5 mm) into a 15% sodium dodecyl sulfate polyacrylamide gel electrophoresis gel. The gel was washed 3x in ddH_2_O for 15 min each and visualized by staining overnight with GelCode® Coomassie blue reagent (Pierce). Stacked protein bands were excised from the gel, reduced with dithiothreitol, and alkylated with iodoacetamide. In-gel digestion was performed using 5 ng/μL mass spectrometry-grade trypsin (Trypsin Gold, Promega) in 50 mM NH_4_HCO_3_ digestion buffer. The resulting peptides were desalted using a Stage Tip manually packed with Empora C18 High-Performance Extraction Disks (3 M)^77^ and eluted peptide solutions were dried under vacuum. Peptides were then resuspended in 18 μL acetonitrile, and 57 μL of 0.2 M HEPES pH 8.5 was added to each sample. TMT10-plex amine reactive reagents (Thermo Fisher, 5 mg per vial) were resuspended in 1024 μL anhydrous acetonitrile and 25 μL of reagent was added to each sample (TMT label: peptide [*w*/*w*] = 12:1) and mixed briefly by vortexing. The mixture was incubated at RT for 1 h, quenched by the addition of 10 μL 5% hydroxylamine for 15 min, and acidified by the addition of 10 μL 10% formic acid. A 5-µL aliquot from each reaction was desalted on a StageTip, analyzed by liquid-chromatography–tandem mass spectrometry (LC-MS/MS) with a Q Exactive Orbitrap HF (high field), and the resulting spectra searched with MaxQuant using its corresponding TMT label as variable modifications on N-terminus and lysine. The percentage of peptides with either N-terminal or lysine TMT labels was calculated, indicating the labeling efficiency for each channel. Labeling efficiency was 96% or greater for each channel. To ensure that equal amounts of labeled peptides from each channel were mixed together, a two-step mixing strategy was employed: in the first step, an identical ~1 μL volume of peptides from each channel was mixed and analyzed, and the value of the median ratio (median of the ratios of all peptide intensities of one channel over their corresponding peptide average intensities of all channels) for each channel is determined as the correction factor. In the second step, the rest of the peptides were mixed by adjusting their volume using the correction factors. In this way, median ratios ranging from 0.97 to 1.02 were achieved as previously reported^78^. The final mixture of reaction products from ten TMT channels were desalted on a Sep-Pak tC18 1 mL Vac Cartridge (Waters, #WAT03820). Eluted peptides were dried by vacuum centrifugation and stored at –20 °C.

### Liquid-chromatography–tandem mass spectrometry

Online chromatography was performed with a Thermo Easy nLC 1000 ultrahigh-pressure UPLC system (Thermo Fisher) coupled online to a Q Exactive HF with a NanoFlex source (Thermo Fisher). Analytical columns (~30 cm long and 75 μm inner diameter) were packed in house with ReproSil-Pur C18 AQ 3 μM reversed-phase resin (Dr. Maisch GmbH, Ammerbuch-Entringen). The analytical column was placed in a column heater (Sonation GmbH, Biberach) regulated to a temperature of 45 °C. The TMT peptide mixture was loaded onto the analytical column with buffer A (0.1% formic acid) at a maximum back-pressure of 300 bar. Peptides were eluted with a 2-step gradient of 3 to 40% buffer B (100% ACN and 0.1% formic acid) in 180 min and 40 to 90% B in 20 min, at a flow rate of 250 nL/min over 200 min using a 1D online LC-MS2 data-dependent analysis (DDA) method as follows: MS data were acquired using a data-dependent top-10 method, dynamically choosing the most abundant not-yet-sequenced precursor ions from the survey scans (300–1750 Th). Peptide fragmentation was performed via higher energy collisional dissociation (HCD) with a target value of 1 × 10^5^ ions determined with predictive automatic gain control. Isolation of precursors was performed with a window of 1 Th. Survey scans were acquired at a resolution of 120,000 at *m*/*z* 200. Resolution for HCD spectra was set to 60,000 at *m*/*z* 200 with a maximum ion injection time of 128 ms. The normalized collision energy was 35. The underfill ratio specifying the minimum percentage of the target ion value likely to be reached at the maximum fill time was defined as 0.1%. Precursor ions with single, unassigned, or seven and higher charge states were excluded from fragmentation selection. Dynamic exclusion time was set at 30 s. Each of the TMT 10-plex samples was analyzed in triplicate.

All data were analyzed with the MaxQuant proteomics data analysis workflow (version 1.5.5.1) with the Andromeda search engine^79,80^. The type of the group specific analysis was set to Reporter ion MS2 with 10-plex TMT as isobaric labels for Q Exactive High Field MS2 data. Reporter ion mass tolerance was set to 0.01 Da, with activated Precursor Intensity Fraction (PIF) value set at 0.75. False discovery rate was set to 1% for protein, peptide spectrum match, and site decoy fraction levels. Peptides were required to have a minimum length of eight amino acids and a maximum mass of 4600 Da. MaxQuant was used to score fragmentation scans for identification based on a search with an allowed mass deviation of the precursor ion of up to 4.5 ppm after time-dependent mass calibration. The allowed fragment mass deviation was 20 ppm. MS2 spectra were used by Andromeda within MaxQuant to search the Uniprot mouse database (01092015; 16,699 entries) combined with 262 common contaminants. Enzyme specificity was set as C-terminal to arginine and lysine, and a maximum of two missed cleavages were allowed. Carbamidomethylation of cysteine was set as a fixed modification and N-terminal protein acetylation, deamidated (N, Q) and oxidation (M) as variable modifications. The reporter ion intensities were defined as intensities multiplied by injection time (to obtain the total signal) for each isobaric labeling channel summed over all MS/MS spectra matching to the protein group as previously validated^80^. Following MaxQuant analysis, the protein and peptide.txt files were imported into Perseus (version 1.5.6.0) software, which was used for statistical analysis of all the proteins identified.

### Identification and analysis of the AIDA-1 interactome

Enrichment for proteins in each 10-plex IP sample was calculated by dividing the relative abundance in the IP by the relative abundance in the appropriate control mouse IgG bead sample (agarose or magnetic) (Supplementary Data 6a). In the agarose bead experiments, analysis of all abundance values showed that the top 5% were above ~1.4-fold enrichment, while the cutoff for the top 5% in magnetic bead experiments was ~3.0-fold. The appropriate cutoff was applied to each IP sample to obtain an enriched population of 255 proteins that were ≥1.4-fold enriched in any agarose bead experiment or ≥3.0-fold enriched in any magnetic bead experiment. Only proteins with three or more peptides identified were included in the analysis, to obtain a reliable mean relative abundance for each protein. The list of proteins enriched in any AIDA-1 IP sample (defined as the AIDA-1 interactome, Supplementary Data 6b) was used as the input for multiple proteins in StringDB under the species *Mus musculus*. Raw data for gene ontology (GO) enrichments are given in Supplementary Data 7, and the protein–protein interaction image (Fig. 4d) was generated by setting the minimum interaction score to 0.150, hiding disconnected network nodes, and displaying connections by strength of supporting data^44^. For functional annotation and network construction in Ingenuity Pathway Analysis (QIAGEN Bioinformatics, version 01-13), the AIDA-1 interactome (Supplementary Data 6b) was used as input for a Core Analysis using the following default settings: Expression Analysis; General Settings = Ingenuity Knowledge Base (Genes Only), Direct and Indirect Relationships; Networks = Interaction networks, Include endogenous chemicals; Node Types = All; Data Sources = All; Confidence = Experimentally Observed; Species = All; Tissues & Cell Lines = All; Mutations = All. The top-scoring network from this analysis (Network 1) is depicted in Fig. 5a. Raw data obtained from the analysis is included as supplementary Supplementary Data 8.

### Statistical analysis

Statistical tests were performed in JMP (version 14, SAS) as in Supplementary Data 4. For AIDA-1 expression by western blot (Fig. 1g and Supplementary Fig. 2c), two-way ANOVA was performed to probe for main effect of Genotype (Unaffected/WT or Proband/Het), Band (AIDA-1 isoform), and Genotype-Band interaction. Although no isoform-specific effects of genotype were detected, two-sided Student’s *t*-tests were performed as indicated. For RT-qPCR expression, two-way ANOVA was performed for main effect of Genotype (Unaffected/WT or Proband/Het), Target (Exon number), and Genotype-Target interaction (Fig. 1h and Supplementary Fig. 2d). Although no exon-specific effects of genotype were detected, one-sided Student’s *t*-tests were performed as indicated. For gross morphological analysis (Fig. 2d), sex differences were expected and two-sided Student’s *t-*tests were performed independently for each sex. For histological analysis of brain regions, two-way ANOVA showed no effects of Genotype, Sex, or Genotype-Sex interaction. Sexes were combined for two-sided Student’s *t-*tests. To analyze specificity of the AIDA-1 interactome, two-sided Fisher’s exact test was performed on the number of proteins enriched in each sample, with the AIDA-1 Mix agarose bead sample as a reference for overlap and the total number of proteins enriched in any sample as the statistical background.

### Reporting summary

Further information on research design is available in the Nature Research Reporting Summary linked to this article.

## Supplementary information

Supplementary Information

Peer Review

Reporting Summary

Description of Additional Supplementary Files

Supplementary Data 1

Supplementary Data 2

Supplementary Data 3

Supplementary Data 4

Supplementary Data 5

Supplementary Data 6

Supplementary Data 7

Supplementary Data 8

## Data Availability

All raw data and the datasets generated during and/or analyzed during the current study are available from the corresponding author on reasonable request.
