# Supplementary material for: Haploinsufficiency in the ANKS1B gene encoding AIDA-1 leads to a neurodevelopmental syndrome
Source: Nat Commun. 2019 Aug 6;10:3529. doi: 10.1038/s41467-019-11437-w (PMC6684583; doi:10.1038/s41467-019-11437-w)
Supplement: Supplementary file 1 — Supplementary Information [file 41467_2019_11437_MOESM1_ESM.pdf]

Haploinsufficiency in the *ANKS1B* Gene Encoding AIDA-1 Leads to a Neurodevelopmental Syndrome

Carbonell et al.

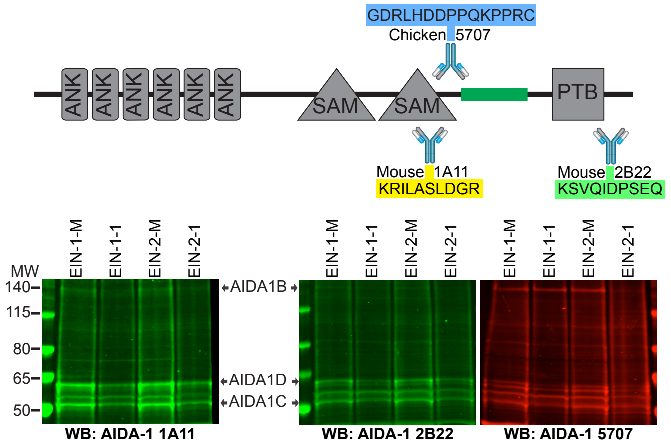

Supplementary Figure 1. **Reduction of AIDA-1 in *ANKS1B* haploinsufficiency syndrome proband-derived neurons using multiple AIDA-1 antibodies**. In both Family EIN-1 and Family EIN-2, antibodies raised to three different peptide sequences detect a reduction of AIDA-1 in proband-derived neurons (EIN-1-1 and EIN-2-1) compared to neurons from unaffected mothers (EIN-1-M and EIN-2-M) by Western blot (10 μg lysate).

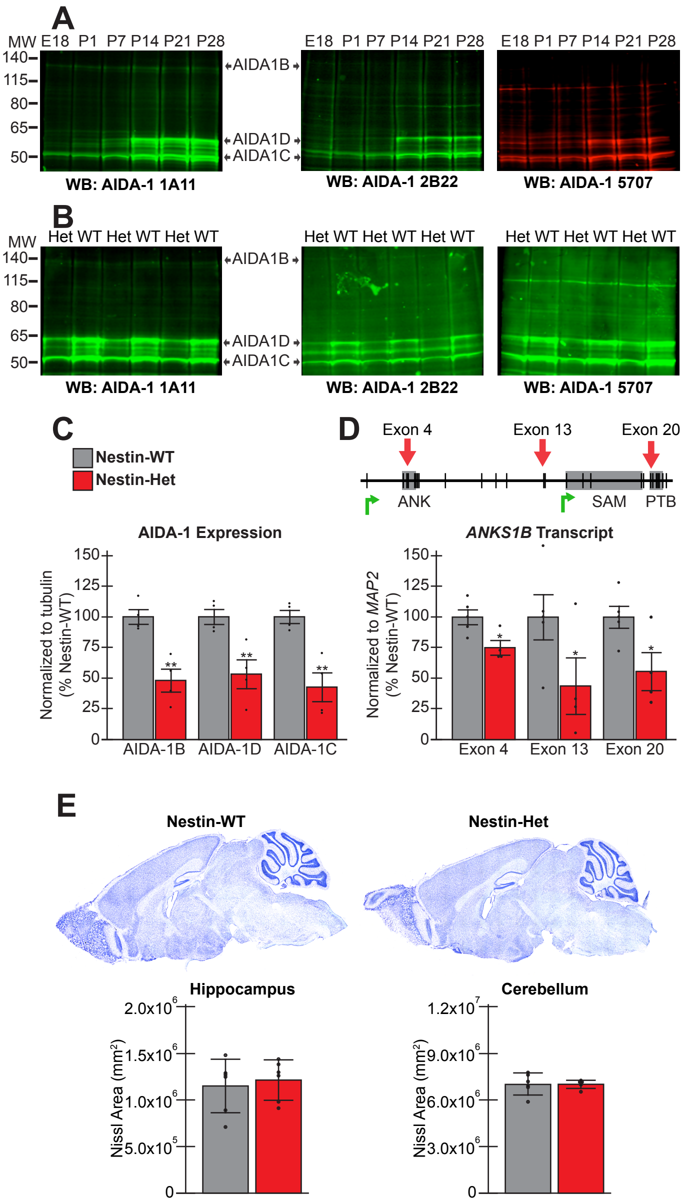

Supplementary Figure 2. *Anks1b* Nestin-Het mice show reduced AIDA-1 expression and no gross anatomical changes in hippocampus and cerebellum. **A**) All three AIDA-1 antibodies detect similar expression of AIDA-1 isoforms in mice throughout development by Western blot (20 µg lysate). **B**) All AIDA-1 antibodies show reduction of AIDA-1 expression in *Anks1b* Nestin-Het compared to Nestin-WT mice by Western blot (20 µg lysate). **C**) Quantitation of AIDA-1 expression by Western blot in Nestin-Het mice shows significant reduction across all major isoforms. *N*=8 mice. Bar graphs show mean ± SEM, two-sided Student's *t*-test, ***p*<0.01. **D**) Quantitation of *Anks1b* expression by RT-qPCR shows significant reduction across exons in Nestin-Het mice. *N*=9 mice. Bar graphs show mean ± SEM, one-sided Student's *t*-test, **p*<0.05. **E**) Nissl staining of sagittal brain sections shows no change in area of hippocampal or cerebellar brain regions in Nestin-Het mice compared to Nestin-WT controls. *N*=12 mice. Bar graphs show mean ± SEM.

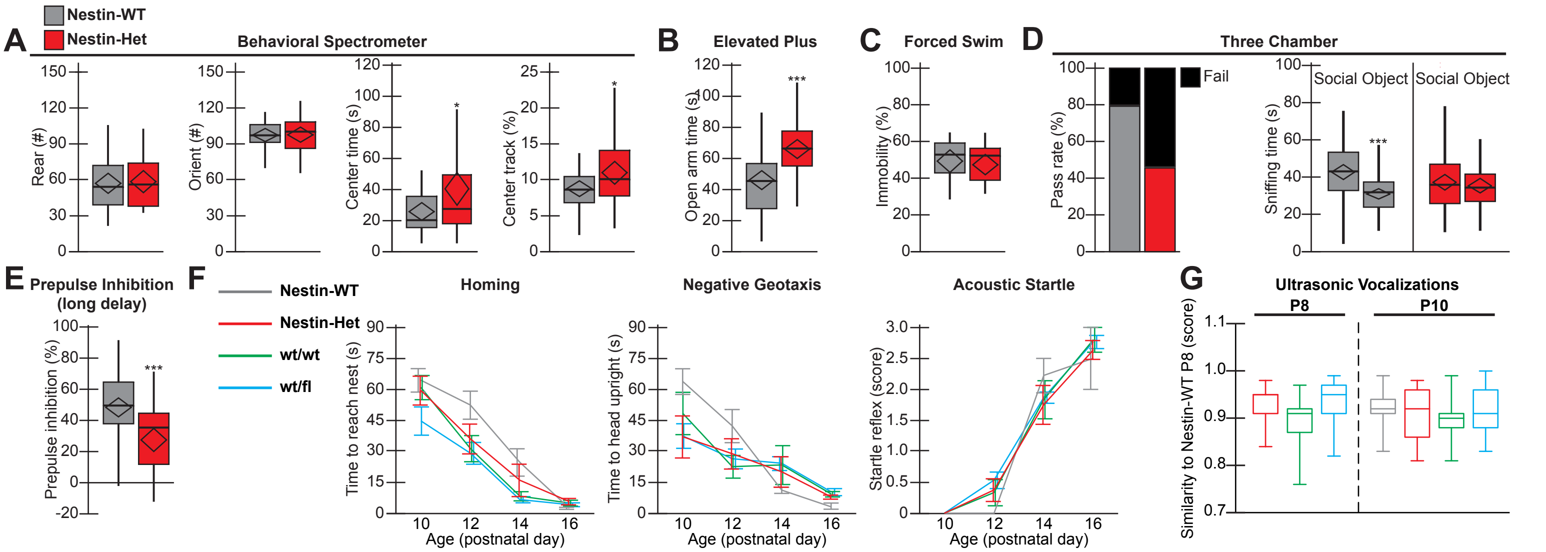

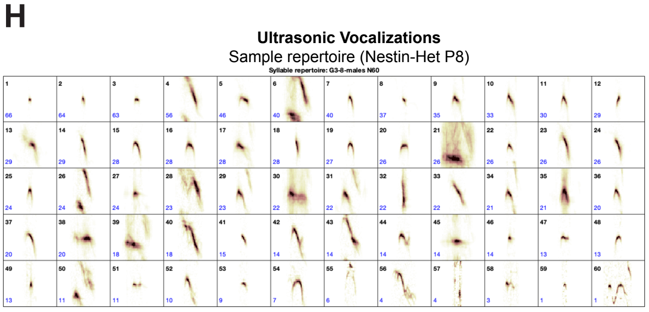

I

		P8					P10			
		Nestin-WT	Nestin-Het	wt/wt	wt/fl		Nestin-WT	Nestin-Het	wt/wt	wt/fl
Power spectral density (kHz)	Mean	74.90	75.95	67.84	73.23		92.42	83.04	72.73	76.47
	Standard deviation	21.72	21.42	18.47	23.63		19.90	25.71	22.82	23.65
Frequency bandwidth (kHz)		43.43	42.84	36.94	47.27		39.81	51.41	45.65	47.31
Syllables per second (#)	Mean	3.07	2.92	2.95	2.94		2.50	2.61	2.93	2.64
	Median	3	3	3	3		2	2	3	2
	Standard deviation	1.81	1.68	1.53	1.61		1.58	1.63	1.61	1.55
Inter-syllable interval (ms)	Mean	114.6	105.7	126.3	120.8		118.7	115.9	114.3	113.4
	Median	129.6	115.2	137.6	131.2		128.0	124.8	120.0	120.0
	Standard deviation	59.8	56.9	53.7	53.8		51.7	53.2	44.6	47.5
Syllable duration (ms)	Mean	50.07	48.49	55.86	53.12		45.44	46.45	54.80	51.36
	Median	48.00	44.80	54.40	49.60		41.60	41.60	52.80	48.00
	Standard deviation	21.82	23.33	23.22	23.95		21.47	21.95	23.86	23.86
Total syllables (#)		4887	4513	8154	7938		2968	2770	6473	5125
Total syllable time (s)		250.2	233.6	507.8	461.4		138.9	132.5	382.2	280.1
Total vocalization time (s)		5038	5044	6837	7589		5039	4683	6840	7211

Supplementary Figure 3. Additional behavioral results from *Anks1b* Nestin-Het mice. A) No differences in rearing or orienting were observed in *Anks1b* Nestin-Het mice compared to Nestin-WT controls. *N*=46 mice. Nestin-Het mice spend significantly more time and track in the center square of the open field as a percentage of total track. *N*=48 mice. **B**) Nestin-Het mice also spend significantly more time in the open arms of the elevated plus maze. *N*=97 mice. **C**) No differences were observed in the Porsolt forced swim test of learned helplessness. *N*=46 mice. **D**) Nestin-Het mice failed the three-chamber test of social approach (Fail = spent <50% of total exploration time with animal) more than Nestin-WT mice. Nestin-WT mice spent significantly more time sniffing the animal (social) than the object, but there was no difference between social and object exploration time in Nestin-Het mice (Tukey Kramer HSD test, ***p<0.001). *N*=97 mice. **E**) Deficit in prepulse inhibition of the acoustic startle reflex was robust and significant at a longer delay (200 ms) between prepulse and startle stimuli. *N*=97 mice. Box plots show the mean and 95% confidence intervals (black diamond), median (black line), 25th-75th quantile (gray or red bar), and range (black whiskers). Student's t-test, *p<0.05 ***p<0.001 except in **D**. **F**) Homing time, negative geotaxis time, and acoustic startle score showed no differences in Nestin-Het pups compared to littermate control groups from P10 to P16. *N*=38 mice (9 Nestin-Het, 8 Nestin-WT, 6 wt/wt, 15 wt/fl). P = postnatal day. Line graphs show mean ± SEM. **G**) Similarity scores of ultrasonic vocalizations (USVs) generated by MUPET. Box plots show median (line), 25th-75th quantile (box), and range (whiskers). **H**) Sample repertoire of USVs from a P8 Nestin-Het pup. **I**) No differences in diverse syllable parameters were observed between Nestin-Het and Nestin-WT pups at P8 or P10. The littermate groups wt/wt (*Anks1b*$^{wt/wt}$) and wt/flox (*Anks1b*$^{wt/fl}$) showed increases in syllable duration and number, total syllable time, and total vocalization time that was not statistically significant. *N*=72 mice (15 Nestin-Het, 15 Nestin-WT, 20 wt/wt, 22 wt/fl).

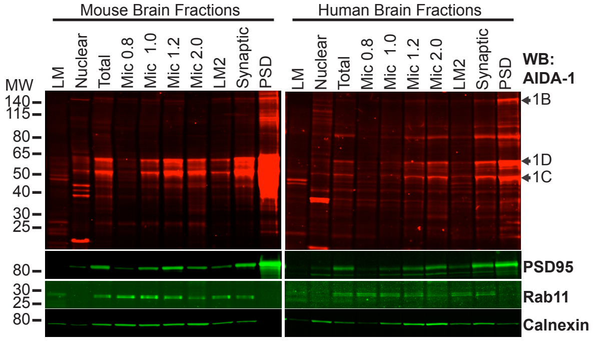

Supplementary Figure 4. Subcellular fractionation of postmortem human and mouse brain. AIDA-1 is enriched in synaptic and postsynaptic density (PSD) fractions, as well as microsomal fractions (Mic 1.2 and 2.0) that are also enriched in recycling endosome marker Rab11 and ER marker calnexin. 20 μg fractions, LM = light membrane fraction.
