# Supplementary material for: Haploinsufficiency in the ANKS1B gene encoding AIDA-1 leads to a neurodevelopmental syndrome
Source: Nat Commun. 2019 Aug 6;10:3529. doi: 10.1038/s41467-019-11437-w (PMC6684583; doi:10.1038/s41467-019-11437-w)
Supplement: Supplementary file 3 — Reporting Summary [file 41467_2019_11437_MOESM3_ESM.pdf]

nature research

Corresponding author(s): Bryen Jordan

Last updated by author(s): Jul 10, 2019

Reporting Summary

Nature Research wishes to improve the reproducibility of the work that we publish. This form provides structure for consistency and transparency in reporting. For further information on Nature Research policies, see Authors & Referees and the Editorial Policy Checklist.

Statistics

For all statistical analyses, confirm that the following items are present in the figure legend, table legend, main text, or Methods section.

n/a	Confirmed	
☐	☒	The exact sample size (*n*) for each experimental group/condition, given as a discrete number and unit of measurement
☐	☒	A statement on whether measurements were taken from distinct samples or whether the same sample was measured repeatedly
☐	☒	The statistical test(s) used AND whether they are one- or two-sided<br>*Only common tests should be described solely by name; describe more complex techniques in the Methods section.*
☐	☒	A description of all covariates tested
☐	☒	A description of any assumptions or corrections, such as tests of normality and adjustment for multiple comparisons
☐	☒	A full description of the statistical parameters including central tendency (e.g. means) or other basic estimates (e.g. regression coefficient) AND variation (e.g. standard deviation) or associated estimates of uncertainty (e.g. confidence intervals)
☐	☒	For null hypothesis testing, the test statistic (e.g. *F*, *t*, *r*) with confidence intervals, effect sizes, degrees of freedom and *P* value noted<br>*Give P values as exact values whenever suitable.*
☒	☐	For Bayesian analysis, information on the choice of priors and Markov chain Monte Carlo settings
☒	☐	For hierarchical and complex designs, identification of the appropriate level for tests and full reporting of outcomes
☒	☐	Estimates of effect sizes (e.g. Cohen's *d*, Pearson's *r*), indicating how they were calculated

Our web collection on statistics for biologists contains articles on many of the points above.

Software and code

Policy information about availability of computer code

Data collection	LI-COR Image Studio, Bio-Rad CFX Manager, Zeiss Axiovision, Zeiss Zen (blue), Biobserve Viewer, SR-LAB software, Avisoft Recorder, MaxQuant
Data analysis	SAS JMP 14, NIH Image J, MATLAB MUPET tool, Perseus, StringDB, QIAGEN Ingenuity Pathway Analysis

For manuscripts utilizing custom algorithms or software that are central to the research but not yet described in published literature, software must be made available to editors/reviewers. We strongly encourage code deposition in a community repository (e.g. GitHub). See the Nature Research guidelines for submitting code & software for further information.

Data

Policy information about availability of data

All manuscripts must include a data availability statement. This statement should provide the following information, where applicable:

- Accession codes, unique identifiers, or web links for publicly available datasets
- A list of figures that have associated raw data
- A description of any restrictions on data availability

All raw data and the datasets generated during and/or analyzed during the current study are available from the corresponding author on reasonable request.

Field-specific reporting

Please select the one below that is the best fit for your research. If you are not sure, read the appropriate sections before making your selection.

☒ Life sciences ☐ Behavioural & social sciences ☐ Ecological, evolutionary & environmental sciences

Life sciences study design

All studies must disclose on these points even when the disclosure is negative.

Sample size	In animal behavior assays, sample sizes were estimated based on power analysis in JMP for least significant number (LSN) in a 2-sided Student's t-test (alpha=0.05) for an effect size and variance observed in a pilot study cohort. Additional cohorts were added to reach LSN and to balance males and females for 2-way ANOVA. For studies with no observed difference, only 1 additional cohort was tested. Retrospective power analysis from JMP and LSN are included in Supplementary Data 5. Sample sizes for Western blots and imaging were chosen based on previous studies that had demonstrated significant effects in 2-sided Student's t-test. Sample sizes for RT-qPCR were sufficient to apply 1-sided Student's t-test given the variance observed in RT-qPCR and the nature of the hypothesis, which was to test for reduced expression.
Data exclusions	No data were excluded from data analyses.
Replication	In human clinical phenotyping, recent records were obtained and a different neuropsychological test was performed for the same domain if applicable: all scores were similar to prior results. For induced neuron experiments, all Ns are given as independent biological replicates from separate inductions. Western blots for AIDA-1 expression were performed using several different antibodies. In animal behavioral assays, mice were tested as 5 independent cohorts to verify reproducibility. For the AIDA-1 interactome, co-immunoprecipitations were repeated and hits confirmed by Western blot. All these attempts at replication were successful.
Randomization	Animals were genotyped at weaning age for adult studies or at P10 for developmental studies, then randomly allocated to mixed-genotype cages until final experimental age. Age was controlled across experimental groups, and mean weight was the same among sexes. Sex was included as a covariate (effect and interaction in ANOVA) when applicable. Covariates for allocation were not relevant to induced neuron experiments: iPSCs were plated in random order and induced simultaneously.
Blinding	Investigators were blinded to experimental groups during data collection and analysis. For human clinical phenotyping, blinding was not possible during clinical interview since medical genetics diagnosis was part of relevant history.

Reporting for specific materials, systems and methods

We require information from authors about some types of materials, experimental systems and methods used in many studies. Here, indicate whether each material, system or method listed is relevant to your study. If you are not sure if a list item applies to your research, read the appropriate section before selecting a response.

Materials & experimental systems

n/a	Involved in the study	
☐	☒	Antibodies
☒	☐	Eukaryotic cell lines
☒	☐	Palaeontology
☐	☒	Animals and other organisms
☐	☒	Human research participants
☒	☐	Clinical data

Methods

n/a	Involved in the study	
☒	☐	ChIP-seq
☒	☐	Flow cytometry
☒	☐	MRI-based neuroimaging

Antibodies

Antibodies used	AIDA-1 (Santa Cruz #376610, clone C-10), tubulin (Thermo Fisher #80017, clone YL1/2), PSD95 (NeuroMab, clone K28/43), GAPDH (Cell Signaling Tech #2118, clone 14C10), calnexin (Genscript #A01240, polyclonal), Rab11 (Cell Signaling Tech #5589, clone D4F5), Git1 (Santa Cruz #365084, clone A-1), Itsn1 (Santa Cruz #136242, clone 29), AP2a1/2 (Santa Cruz #17771, clone C-8), Asap1 (Santa Cruz #374410, clone B-10), Srgap2 (Proteintech #22519-1-AP, polyclonal), GluN2B (Alomone #AGC-003, polyclonal), GluN2A (Alomone #AGC-002, polyclonal), Oct-4 (Abcam #27985, polyclonal), Sox-2 (Cell Signaling Tech #3728, clone C70B1), MAP-2 (EnCor #CPCA-MAP-2, polyclonal). AIDA-1 antibodies 1A11, 2B22, and 5707 were developed and validated in-house.
Validation	Each primary antibody was validated for the species and application by noting validation statements on the manufacturer's website. In-house AIDA-1 antibodies are validated in the manuscript by shRNA knockdown and in the supplementary information by heterozygous mouse knockout.

Animals and other organisms

Policy information about studies involving animals; ARRIVE guidelines recommended for reporting animal research

Laboratory animals	All animal studies used the species Mus musculus, strain B6.Cg-Tg(Nes-cre)1Kln/J (Jackson Laboratories stock #003771) backcrossed to strain C57BL/6J (Jackson Laboratories stock #000664) and crossed to the Anks1b-floxed strain on the C57BL/6J background. For developmental behavioral assays, male and female pups 8-16 days old were used. For morphometry and adult

	behavioral assays, male and female mice 3-4 months of age were used. For Western blot and RT-qPCR, 6-month-old female mice were used. For fractionation and co-immunoprecipitation, 2-month-old male mice from the C57BL/6J strain were used.
Wild animals	The study did not involve wild animals.
Field-collected samples	The study did not involve samples collected from the field.
Ethics oversight	All experiments complied with all relevant ethical regulations for animal testing and research, and were approved by the Albert Einstein College of Medicine Institutional Animal Care and Use Committee (IACUC).

Note that full information on the approval of the study protocol must also be provided in the manuscript.

Human research participants

Policy information about studies involving human research participants

Population characteristics	Covariate population characteristics were not relevant since no comparisons were made between groups and no adjustments were necessary.
Recruitment	Participants for phenotyping, genotyping, and IPSC generation were recruited by direct contact by researchers. Self-selection is not likely to impact these results since clinical phenotyping utilized population-standardized measures, and all protocols were performed blind to genotype. Recruitment of additional patients through Autism Speaks, DECIPHER, and GeneMatcher may select for probands with developmental phenotypes since these resources are used by clinicians and researchers studying abnormal genotypes and phenotypes.
Ethics oversight	Clinical phenotyping received ethical approval by the Institutional Review Board (IRB) at Albert Einstein College of Medicine in IRB protocol #2011-320 to SM. Whole-exome sequencing and IPSC generation received ethical approval by the Institutional Review Board (IRB) at Albert Einstein College of Medicine in IRB protocol #2017-8311 to BAJ.

Note that full information on the approval of the study protocol must also be provided in the manuscript.
