# Supplementary material for: Haploinsufficiency in the ANKS1B gene encoding AIDA-1 leads to a neurodevelopmental syndrome
Source: Nat Commun. 2019 Aug 6;10:3529. doi: 10.1038/s41467-019-11437-w (PMC6684583; doi:10.1038/s41467-019-11437-w)
Supplement: Supplementary file 4 — Description of Additional Supplementary Files [file 41467_2019_11437_MOESM4_ESM.docx]

**Description of Additional Supplementary Files**

File Name: Supplementary Data 1 Detailed clinical phenotyping of *ANKS1B* deletion probands from families EIN-1 and EIN-2.
Description: Comprehensive clinical interviews and examination revealed developmental delays and deficits in motor coordination. ADHD = attention deficit/hyperactivity disorder. For the general sensory motor exam, Yes = ability to perform task within normal limits and No = difficulty or inability to perform task. Neuropsychological tests were administered and scored for each subject’s developmental age. Wechsler scales and subscales included the following: Wechsler Primary Preschool Scale of Intelligence, Fourth Edition (WPPSI-IV); Wechsler Scale of Intelligence for Children, Fifth Edition (WISC-V); and Wechsler Adult Intelligence Scale, Fourth Edition (WAIS-IV). WIAT-III = Wechsler Individual Achievement Test, Third Edition. BNT = Boston Naming Test. NEPSY-II = Developmental NEuroPSYchological Assessment, Second Edition. CELF-V = Clinical Evaluation of Language Fundamentals, Fifth Edition. Movement ABC = Movement Assessment Battery for Children. VMI- VI = Beery Buktenika Developmental Test of Visual-Motor Integration, Sixth Edition. RCFT = Rey Complex Figure Test. CVLT-C = California Verbal Learning Test, Children’s Version. CVLT-II = California Verbal Learning Test, Second Edition. CPT-III = Conners’ Continuous Performance Test, Third Edition. K-CPT-II = Conners’ Kiddie Continuous Performance Test, Second Edition. D-KEFS = Delis-Kaplan Executive Function System (D-KEFS). SDMT = Symbol Digit Modalities Test. ADOS-II = Autism Diagnostic Observation Schedule, Second Edition. SRS-II = Social Responsiveness Scale, Second Edition. Vineland-III = Vineland Adaptive Behavior Scales, Third Edition. Tests generated a standard score (mean = 100, standard deviation = 15), T score (mean = 50, standard deviation = 10), scaled score (mean = 10, standard deviation = 3), *z* score (mean= 0, standard deviation = 1), or percentile (%ile) normed for age.

File Name: Supplementary Data 2 Additional *ANKS1B* deletion probands from the DECIPHER database display similar neurodevelopmental phenotypes.
Description: Monogenic *ANKS1B* microdeletion probands report developmental delays, speech and language impairments, cognitive/mental abnormalities, and/or obesity although we did not receive additional data from clinicians. For probands with additional copy-number variants (CNVs) in other genes, clinicians report that patients consistently display intellectual disability (ID), speech and motor delays, MRI abnormalities, and autism spectrum disorder (ASD) or attention-deficit hyperactivity disorder(ADHD). No microcephaly or macrocephaly was reported. DEC = DECIPHER database, DD = developmental delay, MRI = magnetic resonance imaging, EEG = electroencephalography.

File Name: Supplementary Data 3 Predicted high-impact single nucleotide variants (SNVs) segregating with *ANKS1B* microdeletion in families EIN-1 and EIN-2
Description: Whole exome sequencing was performed for *ANKS1B* deletion probands EIN-1-1 and EIN-2-1, along with their respective affected fathers (EIN-1-4 and EIN-2-3) and unaffected mothers (EIN-1-M and EIN-2-M). SNVs that are predicted to highly affect gene expression, annotated in the ClinVar database, and segregated with the *ANKS1B* deletion and phenotype are listed.

File Name: Supplementary Data 4
Description: Statistical analysis for Figures 1, 2, 4, 5, and Supplementary Figure 2. For Western blot of AIDA-1 expression Figure 1F, statistical analysis was not performed for family EIN-1because a single control sample was loaded on each gel (*N*=3-5 gels). For NMDAR subunit surface expression in Figure 5C, 1-3 regions of interest were analyzed for each of 60-99 neurons. All statistical analysis was performed in JMP version 14 (SAS).

File Name: Supplementary Data 5 Statistical analysis for behavioral tests in the *Anks1b* heterozygous mouse model (Figure 3 and Supplementary Figure 3)

Description: For all adult tests, 2-way ANOVA for main effect of Genotype, Sex, and Genotype-Sex interaction was performed. Tests showing significant main effect of Genotype (*α*=0.05) were probed using post hoc Student’s *t*-tests. Sample size was evaluated using least significant number (LSN) and retrospective power analysis. For the three-chamber test, likelihood ratio effect tests (with post hoc chi-squared tests and Fisher’s exact test) and repeated measures ANOVA (with post hoc Tukey Kramer HSD test) were additionally performed. All statistical analyses were performed in JMP version 14 (SAS).

File Name: Supplementary Data 6

Description: A) Quantification of all proteins identified by TMT-MS in all 10 immunoprecipitations. Values for each protein in column D-K were calculated by dividing the abundance in each of the 8 IPs by the abundance in the corresponding IgG control bead (agarose or magnetic). Analysis of these values showed that the top 5% of values in each bead methodology were **1.4 (agarose) and **3.0 (magnetic). Proteins X and Y are synaptic proteins used as control IPs. B) The AIDA-1 interactome. We defined the AIDA-1 interactome as the 163 proteins with >2 peptides identified that are enriched >1.4 fold (agarose) or >3.0 fold (magnetic) above control IgG bead samples in any of the four AIDA-1 immunoprecipitations (IPs). Genes are ordered first by number of AIDA-1 IPs in which they are enriched, then by their fold enrichment in the AIDA-1 Mix Agarose IP.

# File Name: Supplementary Data 7

# Description: A) Gene ontology (GO) terms in Cellular Components significantly enriched in the AIDA-1 interactome from StringDB. B) Gene ontology (GO) terms in Biological Processes significantly enriched in the AIDA-1 interactome from StringDB. C) Gene ontology (GO) terms in Molecular Function significantly enriched in the AIDA-1 interactome from StringDB.

File Name: Supplementary Data 8
Description: A) Top Disease and Function annotations significantly enriched in the AIDA-1 Interactome from Ingenuity Pathway Analysis (QIAGEN Bioinformatics). B) Integrated Analysis of the AIDA-1 Interactome from Ingenuity Pathway Analysis (QIAGEN Bioinformatics). C) Top Networks in the AIDA-1 interactome identified by Ingenuity Pathway Analysis (QIAGEN Bioinformatics).
