# Supplementary material for: Haploinsufficiency in the ANKS1B gene encoding AIDA-1 leads to a neurodevelopmental syndrome
Source: Nat Commun. 2019 Aug 6;10:3529. doi: 10.1038/s41467-019-11437-w (PMC6684583; doi:10.1038/s41467-019-11437-w)
Supplement: Supplementary file 2 — Peer Review [file 41467_2019_11437_MOESM2_ESM.pdf]

Reviewers' comments:

Reviewer #1 (Remarks to the Author):

This is an interesting paper trying to show an association between anks1b and neurodevelopmental phenotype. The authors used human cell data, mouse knockout, and a new mass spec technique to investigate this association. The manuscript is well written.

Major points:

The authors mention that the presence of a mutation may be not consistent with viability and yet there is a father with an inherited mutation in the specified gene.

The authors made an effort to rule out exomic variance. Still the link between CNV and disease is not overwhelming.
What is the frequency of this CNV in the ExAC database?

Fig 1 E, F:
The iN section of this paper appears somewhat superficial
If the authors wish to include this section there are basic standards that one expects to see. For example, 2 of 20 cells only show MAP2. This would suggest the differentiation protocol is inefficient or that neurons are not solely generated.
The WB has no loading control that we normally expect.
In general, this section is below the standards one would expect which makes it difficult to adequately review.

Fig2 A, B, C:
Can the authors please address the rationale for loading controls chosen and the reason for why it is so variable in Fig 2B.
The loading control is also highest in the brain and overall makes it difficult to interpret the results of the WB.

Fig 4 C:
It would be advisable to explain the rationale for choice of statistical test. Specifically, why a students t-test has been chosen as opposed to a hypergeometric statistical test.

Can the authors please explain why there is a 52.6 overlap with protein X which we assume acts as a negative control, while there is only a max of 50% overlap. I note that the p-value is not significant for protein X, while it is significant for AIDA-1-2B22.

The authors have mentioned numerous times in the article the NMDAR hypothesis, that NMDA distribution may be mediated by AIDA-1. It would be nice to see evidence of this association.

The mass spec was a nice idea and an involved experiment, yet the outcome of this was essentially a GO analysis. It seems like the full potential of the experiment has not been maximized.

Fig 5A:
The statistical method for constructing the network is not identified which makes it difficult to interpret.

The data used in Fig 5 is the Interactome from figure 4 (we assume). The discussion mentions that the bioinformatics analyses allows them to look at the convergence between healthy and disease.
Were the Nes-Het animals used in the data for Fig 5?

The authors identified a potential synaptic gene related to neurodevelopment. More direct or functional work would supplement the paper by adding electrophysiology work or LTP analyses in mice.

Reviewer #2 (Remarks to the Author):

In this study, Carbonell et al found new patients carrying CNVs overlapping ANKS1B gene region and suggested that ANKS1B haploinsufficiency is a direct cause for neurodevelopmental syndrome by using induced neurons derived from human patients and by characterizing phenotypes of ANKS1B conditional heterozygous mice. They also tried to elucidate mechanistic insights underlying symptoms by quantative proteomic analysis of interactome analysis. Overall, this study strengthened clinical implication of ANKS1B mutations with a neurodevelopmental syndrome. However, to make this study more reliable and solid, there are several things to be solved.

Major issues:
In figure 1A, all deletions were located before SAM domain. Why are the deletions in human patients positioned like that?
In figure 1A, newly found CNV mutations were described. How do you think the N-term mutations decrease all AIDA-1 isoform levels in induced neuron culture although short isoform transcripts were unlikely to be affected by the mutations?
According to figure 1F, the reduction of AIDA-1 expression level seems to be observed by an antibody that may target c-terminal of the protein. However, the micro-deleted ANKS1B gene still have a potential to generate truncated premature forms of the long transcript which express abnormal peptides. These peptides could have gain-of-function or dominant negative effect. Therefore, the decreased signals with c-term antibody would not be enough to determine whether the 'haploinsufficiency' of AIDA-1 leads to syndromic symptoms. I am wondering that the truncated mRNAs undergo nonsense-mediated mRNA decay in induced neurons from human patients as well as in Nestin-Het brain. Practically, RT-qPCR targeting various regions (N-term, middle regions and short isoform-specific sites) should be performed to measure the amount of mRNA of induced neurons in patient and control.
Different exon deletions can cause different transcript expression patterns in case of two different Shank2 knockout mouse lines (exon 6+7 del vs exon 7 del). Different from the N-term microdeletion in human patients, the target region for floxing is PTB domain in mice. This discrepancy makes mouse model less represent human patients. To rule out this case, RT-qPCR abovementioned again would be a good experiment.
In figure 2D, the brain size of Nestin-Het mice was decreased. Is there any case of microencephaly in human case? Moreover, AIDA-1 seems to be expressed highly in hippocampus and cerebellum, which could be more susceptible to ADIA-1 deficiency. Is there any gross morphological changes in those brain regions?
In figure 3, All behavioral data were produced from adult mice. However, all human patients reported in this study have severe behavioral symptoms at very young age. To check whether the behavioral impairments relevant to social interactions and communications can be observed even in young age in mice, pup ultrasonic vocalization test, maternal homing behavior or juvenile play behavior should be performed.
In figure 3C, I am wondering how the social preference was calculated. Raw values such as time spent in chamber and social vs object sniffing time should be presented. Moreover, 'social sniffing time vs object sniffing time' and 'Time spent in social chamber vs Time spent in object chamber' should be statistically compared to determine sociability in mice.
In figure 3D, PPI is performed I cannot find the length of 'long delay'.
In figure 3F, I cannot find how the preference for new is calculated.
In figure 3G, the number of male and female mice should be informed. The number of each gender

should be balanced to prevent biased results.
In figure 4, many interesting interactions were detected in quantitative proteome analysis. To strengthen molecular mechanistic insight, these interactions should be validated by Co-IP or Pull-down assay.

Minor issues:
In figure 1A, it would be better to indicate alternative transcription start site. Some deletions can still let the short transcripts made.
Many western blot data do not show tubulin as control.

We would like to thank the reviewers for their valuable input. We have worked hard to address every concern raised, and feel that the substantial number of added experiments significantly strengthens our manuscript. In this improved study, we have: 1) Clarified all statistical concerns raised by reviewers, 2) Added patient information on microcephaly, 3) Performed additional Western blots for diverse housekeeping proteins to address loading concerns, 4) Carried out RT-qPCR for exons across the gene to corroborate our claims of haploinsufficiency and confirm the construct validity of our animal model, 5) Performed histological analysis to test for brain abnormalities in our animal model, 6) Tested for ultrasonic vocalizations, homing, startle, and negative geotaxis in early development, and tested an additional adult cohort to further balance sex in behavioral assays, 7) Validated novel AIDA-1 interactors identified in our quantitative proteomic assay that are involved in vesicle transport and neural development, and finally 8) Tested a molecular mechanism predicted by our bioinformatic analyses to support the hypothesis that altered NMDAR subunit distribution underlies *ANKS1B* haploinsufficiency syndrome. Please note that all changes to the text have been labeled in red.

We are excited to present this work to the neuroscience community, as it is the first to describe individuals around the globe harboring monogenic heterozygous deletions in the *ANKS1B* gene and displaying neurodevelopmental phenotypes including autism, ADHD, speech apraxia, and motor impairments. Studying this genetic syndrome with confirmed loss of function will allow us to clearly link *ANKS1B* to behavioral and cellular phenotypes, thus providing broad insight into the pathobiology of neurodevelopmental disorders. Below are detailed responses to all concerns raised by Reviewer #1 and Reviewer #2.

Reviewer #1

1) "The authors mention that the presence of a mutation may be not consistent with viability and yet there is a father with an inherited mutation in the specified gene."

We apologize if this was not clear. All patients identified so far are heterozygous for their respective microdeletion. We stated in the manuscript that homozygous deletion in mice is not consistent with viability since homozygous *Nestin-Cre;Anks1b*$^{fl/fl}$ conditional knockout mice do not survive postnatal day 0. Since heterozygous mice are viable, these were used for the morphological and behavioral analysis in this manuscript.

2) "The authors made an effort to rule out exomic variance. Still the link between CNV and disease is not overwhelming. What is the frequency of this CNV in the ExAC database?"

While the term "overwhelming" is subjective, the 14 individuals belonging to 12 different families from around the world (France, the Netherlands, Israel, Canada, and the United States) presented in this study comprise a substantially larger population than most first studies characterizing a novel monogenic neurodevelopmental disorder. ExAC only lists the population frequency of single nucleotide variations (not microdeletions) and the microdeletions we have identified are unique for each respective family. However, we stated in the manuscript that from the ExAC database, *ANKS1B* has a computed pLI (probability of loss-of-function intolerance) of 0.99, indicating that fewer than 10% of the expected protein-truncating variants are observed for this particular gene. Together with analysis of local missense constraints from ExAC, these results show that variation in *ANKS1B* is strongly selected against in the population, suggesting that this gene regulates essential organismal functions.

3) "Fig 1 E, F: The iN section of this paper appears somewhat superficial. If the authors wish to include this section there are basic standards that one expects to see. For example, 2 of 20 cells only show MAP2. This would suggest the differentiation protocol is inefficient or that neurons are not solely generated.

We apologize if this was section was not clear. The additional cells visible in **Figure 1E** are the rat astrocytes added to these human neuronal cultures to improve viability and maturation, and we have added text in the figure legend to clarify this. We have simply chosen an image of isolated neurons to show a sample of neuronal morphology. *Ngn2*-mediated direct-to-neuron conversion of iPSCs (Zhang et al., 2013, *Neuron*) is indeed known to be variable, but a puromycin selection step in this protocol kills most non-stably infected iPSCs. Importantly, the added rat astrocytes do not express AIDA-1, and the Western blot quantification in **Figure 1G** was normalized to the mature neuronal marker PSD95 to account for variable neuronal conversion among samples. During this revision, we added three more replicates to **New Figure 1G** and find that two

additional AIDA-1 specific antibodies show similar decreases in AIDA-1 expression in proband neurons (**New Figure S1**).

4) "The WB has no loading control that we normally expect. In general, this section is below the standards one would expect which makes it difficult to adequately review."

This was an unfortunate oversight as we failed to mention that AIDA-1 levels in Western blots shown in **Figure 1G** had been normalized to the neuronal marker PSD95. We have clarified this in the figure legend and performed additional biological replicates of the experiment: we now show Western blots for AIDA-1, the neuronal marker PSD95, and two additional loading controls (tubulin and GAPDH) for qualitative confirmation of loading in **New Figure 1F**.

5) "Fig2 A, B, C: Can the authors please address the rationale for loading controls chosen and the reason for why it is so variable in Fig 2B. The loading control is also highest in the brain and overall makes it difficult to interpret the results of the WB."

Traditional loading controls such as tubulin and β-actin can vary significantly across different tissues, stages of development, and normal or pathological states (see Mortiz, 2017, *Proteomics* for a review). We had used c-Jun as a loading control in Figure 2A because we had observed that its expression is relatively stable in total brain lysates across development. However, we have repeated these experiments and present two different loading controls (GAPDH and tubulin) for Western blots showing AIDA-1 levels during development (**New Figure 2A**), across tissues (**New Figure 2B**), and between *Anks1b* wildtype and heterozygous mice (**New Figure 2C**). Note the differences between tubulin and GAPDH signal in **New Figure 2B** despite carefully loading 20 ug of protein lysate for each tissue analyzed.

6) "Fig 4 C: It would be advisable to explain the rationale for choice of statistical test. Specifically, why a students t-test has been chosen as opposed to a hypergeometric statistical test.

We apologize if this was not clear, but the statistical test presented is a Fisher's exact test and not a Student's *t*-test and we have clarified this in the text. Fisher's exact test indeed uses the hypergeometric distribution to calculate deviation from the null hypothesis. However, the hypergeometric test obtains the probability of obtaining a single observation, which in this case is the observed totals and number of overlaps between each interactome. Fisher's exact test calculates the sum of the probabilities of obtaining this observation and all other, more extreme observations. Therefore, it yields a true p value by definition (cumulative, not just a single probability) and is more rigorous than a hypergeometric test for this analysis.

7) "Can the authors please explain why there is a 52.6 overlap with protein X which we assume acts as a negative control, while there is only a max of 50% overlap. I note that the p-value is not significant for protein X, while it is significant for AIDA-1-2B22."

We considered that the best possible controls for this experiment would be 1) background proteins pulled down with mouse IgG (to yield fold enrichment over background) and, 2) the interactomes of unrelated synaptic proteins X and Y. Since synapses are tightly packed protein structures (especially in the postsynaptic density), we expected some overlap between these interactomes. The lack of significance for Protein X is because this interactome had a higher number of identified interactors and thus has an increased probability of random overlap, resulting in a higher p value in Fisher's exact test.

8) "The authors have mentioned numerous times in the article the NMDAR hypothesis, that NMDA distribution may be mediated by AIDA-1. It would be nice to see evidence of this association. The mass spec was a nice idea and an involved experiment, yet the outcome of this was essentially a GO analysis. It seems like the full potential of the experiment has not been maximized."

We agree with the reviewer that a test of the NMDAR hypothesis would strengthen this manuscript and add a mechanistic component to our findings. Based on this suggestion, on our previous publication linking AIDA-1 to GluN2B surface expression in mice (Tindi et al., 2015, *J Neuroscience*), and on our interactome analyses implicating AIDA-1 in vesicle function, we measured cell surface levels of the NMDAR subunits GluN2A and

GluN2B in iPSC-derived neurons from patients. Consistent with our previous results, we found a significant increase in GluN2A surface expression, which is shown in **New Fig. 5C**. Contrary to what we expected, we did not find any changes in GluN2B. This discrepancy could be due to species-specific regulation of GluN2B, or to dose-dependent regulation by AIDA-1 since the heterozygous deletions observed in patients result in ~50% residual AIDA-1 and our previous work was performed in homozygous forebrain knockout mice. Additionally, current assays measured total cell surface expression, not specifically at synapses. Unfortunately, synapse formation in our cultures was highly variable, which decreased our confidence for measuring NMDAR subunit expression at synapses. Despite these differences, we show that GluN2A surface levels are altered in proband-derived neurons, which supports the hypothesis that altered NMDAR function is a mechanism of disease.

In addition to these positive results, we are actively exploring other cellular pathways identified by the AIDA-1 interactome. We hope the reviewer acknowledges that the significance of this work lies in our 1) description of patients with a novel neurodevelopmental syndrome whom we have deeply phenotyped, 2) confirmation of haploinsufficiency using newly generated patient-derived neurons, 3) generation of a novel mouse model of the disease with confirmed construct and face validity, 4) presentation of AIDA-1-linked molecular pathways using a novel proteomic approach, and finally 5) identification of an NMDAR deficit that may underlie *ANKS1B* haploinsufficiency syndrome. Unlike studies with a highly reductionist view of gene/protein function, we believe there is great value in presenting a broad, unbiased view of mechanisms that may be dysregulated in *ANKS1B* syndrome.

9) "Fig 5A: The statistical method for constructing the network is not identified which makes it difficult to interpret."

The input criteria for the AIDA-1 interactome and the parameters set for generating the networks in Ingenuity Pathway Analysis (IPA) have been detailed in the **New Methods** section under “Identification and Analysis of the AIDA-1 Interactome”. The IPA Network Generation Algorithm is available from QIAGEN.

10) "The data used in Fig 5 is the Interactome from figure 4 (we assume). The discussion mentions that the bioinformatics analyses allows them to look at the convergence between healthy and disease. Were the Nes-Het animals used in the data for Fig 5?"

The reviewer is correct in that the same AIDA-1 interactome list was used to construct **Figure 4D** in StringDB and as the input for IPA in **Figure 5**. The convergence we describe is the convergence of all the significant functional annotations obtained from IPA (**Table S8a**) on specific categories of diseases, systems, and functions in IPA (**Figure 5A**). This hierarchical structure of ontology in IPA provides more depth than a simple GO analysis and allows us to identify potential roles for AIDA-1 in normal development (functions and systems) and pathological states (diseases and disorders). The co-immunoprecipitations used to generate the interactome data were performed on wild-type mice. These points have been clarified in the text.

11) "The authors identified a potential synaptic gene related to neurodevelopment. More direct or functional work would supplement the paper by adding electrophysiology work or LTP analyses in mice."

We have already shown that loss of AIDA-1 leads to deficits in synaptic function and long-term potentiation in rodent hippocampus using forebrain specific (CaMKIIa-cre) AIDA-1 knockout mice (Tindi et al., 2015, *J Neurosci*). We fully agree with the reviewer that elucidating the role of AIDA-1 in synaptic function will be essential to understand the etiology of *ANKS1B* haploinsufficiency syndrome. However, we do not know what specific brain regions underlie the neurodevelopmental phenotypes present in *ANKS1B* probands and behavioral deficits in Nestin-Het mice. Importantly, neither the Nestin-Het (**Figure 3F)** nor the CaMKIIa-KO mouse model (unpublished) show hippocampus-dependent cognitive deficits in the object placement test. Moreover, intellectual disability is not a common phenotype in *ANKS1B* haploinsufficiency syndrome (**Table 1**). Therefore, in addition to having already performed these experiments, testing plasticity in the hippocampus would lead to results of uncertain relevance to the disease. Although we currently have no compelling scientific rationale for selecting any specific brain region for electrophysiological characterization, we are actively searching for disease-relevant brain structures using genetic and viral targeting.

Reviewer #2

12) "In figure 1A, all deletions were located before SAM domain. Why are the deletions in human patients positioned like that?"

We present all the microdeletions in *ANKS1B* that we have identified in patients so far and do not know why the deletions are positioned in such a manner. However, our analysis of the Exome Aggregation Consortium (ExAC) database shows that missense variations in N-terminal region of *ANKS1B* overlapping many of the deletions found in patients are strongly selected against in the population. This suggests that these regions, which encode the ankyrin repeats in AIDA-1, are crucial for normal function. We note that these are also the regions unique to AIDA-1B, which is present throughout development in mice.

13) "In figure 1A, newly found CNV mutations were described. How do you think the N-term mutations decrease all AIDA-1 isoform levels in induced neuron culture although short isoform transcripts were unlikely to be affected by the mutations?"

Like the reviewer, we were surprised to find that N-terminal microdeletions in families EIN-1 and EIN-2 (**New Figure 1A**) that should have only affected larger AIDA-1 isoforms, also reduced the expression of shorter isoforms whose putative transcription start sites were spared. While we do not know how this occurs, we speculate that the loss of unidentified cis-acting regulatory elements in the deleted regions (which can be over 1 Mbp away from transcription start sites) lead to impaired expression of downstream AIDA-1 isoforms. Alternatively, AIDA-1 isoforms may stabilize each other at the protein level, such that loss of AIDA-1B leads to the degradation of remaining smaller isoforms. We have added these important points to the discussion.

14) "According to figure 1F, the reduction of AIDA-1 expression level seems to be observed by an antibody that may target c-terminal of the protein. However, the micro-deleted ANKS1B gene still have a potential to generate truncated premature forms of the long transcript which express abnormal peptides. These peptides could have gain-of-function or dominant negative effect. Therefore, the decreased signals with c-term antibody would not be enough to determine whether the 'haploinsufficiency' of AIDA-1 leads to syndromic symptoms. I am wondering that the truncated mRNAs undergo nonsense-mediated mRNA decay in induced neurons from human patients as well as in Nestin-Het brain. Practically, RT-qPCR targeting various regions (N-term, middle regions and short isoform-specific sites) should be performed to measure the amount of mRNA of induced neurons in patient and control... Different exon deletions can cause different transcript expression patterns in case of two different Shank2 knockout mouse lines (exon 6+7 del vs exon 7 del). Different from the N-term microdeletion in human patients, the target region for floxing is PTB domain in mice. This discrepancy makes mouse model less represent human patients. To rule out this case, RT-qPCR abovementioned again would be a good experiment."

We thank the reviewer for this insight, which led to considerable thought about our claims of haploinsufficiency. We agree that there is the potential for abnormal splicing and that resulting transcripts could lead to the translation of peptides that may contribute to disease. We have therefore followed the reviewer suggestion and performed RT-qPCR of N-terminal, middle, and C-terminal regions along the *ANKS1B* gene in patient and control neurons (**New Figure 1H**), as well as in *Anks1b* Nestin-Het and Nestin-WT mice to assess the construct validity of our disease model (**New Figure S2D**). We found a reduction in RT-qPCR targets in all three regions in patient compared to control, and in Nestin-Het compared to Nestin-WT, with no target-specific effects of genotype in 2-way ANOVA in either disease model (**New Table S4**).

In addition, we repeated Western blots to measure AIDA-1 expression in human neurons (**New Figure S1A**) and animal model (**New Figure S2B**) using three different antibodies targeting three different regions of the AIDA-1 protein. While all antibodies target C-terminal regions of AIDA-1, we believe that together with the RT-qPCR data, these new results corroborate our claims of haploinsufficiency because the C-terminal areas are the only regions intact following N-terminal deletions.

15) "In figure 2D, the brain size of Nestin-Het mice was decreased. Is there any case of microencephaly in human case?"

Head circumference (HC) is variable among the patients for whom we have this information. Two patients indeed met criteria for microcephaly (HC <3rd percentile), but four other patients scored in the 50th to 95th percentile for HC. For reference, macrocephaly is designated as HC >98th percentile. This data has been added to **New Table 1**.

16) "Moreover, AIDA-1 seems to be expressed highly in hippocampus and cerebellum, which could be more susceptible to ADIA-1 deficiency. Is there any gross morphological changes in those brain regions?"

As suggested, we have compared the anatomy of hippocampal and cerebellar regions in *Anks1b* Nestin-Het mice compared to Nestin-WT mice. We do not observe any gross anatomical abnormalities in these regions and report results in **New Figure S2D**. We show that there are no main effects of Genotype or Genotype-Sex interaction on hippocampal or cerebellar area in **New Table S4**.

17) "In figure 3, All behavioral data were produced from adult mice. However, all human patients reported in this study have severe behavioral symptoms at very young age. To check whether the behavioral impairments relevant to social interactions and communications can be observed even in young age in mice, pup ultrasonic vocalization test, maternal homing behavior or juvenile play behavior should be performed."

We thank the reviewer for this suggestion and have now tested maternal homing (social), acoustic startle (sensory), and negative geotaxis (motor) in mouse pups aged P10 to P16 (**New Figure S3F**). Moreover, we recorded and analyzed pup ultrasonic vocalizations (USVs) in several litters of *Anks1b* Nestin-Het pups and littermate controls. We used an unsupervised and unbiased machine learning based algorithm (MUPET- Van Segbroek et al., 2017, *Neuron*) to compare syllable characteristics, as well as diverse parameters of pup vocalizations at P8 and P10 (**New Figure S3G**). We see no significant differences among genotypes in any domain, suggesting that while the adult Nestin-Het mice recapitulate most phenotypes associated with *ANKS1B* syndrome, these changes emerge post-weaning age.

18) "In figure 3C, I am wondering how the social preference was calculated. Raw values such as time spent in chamber and social vs object sniffing time should be presented. Moreover, 'social sniffing time vs object sniffing time' and 'Time spent in social chamber vs Time spent in object chamber' should be statistically compared to determine sociability in mice."

Social preference was calculated using the following formula: *social preference = (social sniffing time) / (social sniffing time + object sniffing time) x 100%*. This has been added to the new Methods sub-section "**Animal Behavioral Assays**." As suggested, we have added a new graph comparing raw social vs object sniffing time in **New Figure S3D**. Using within-subjects repeated measures ANOVA, we find a significant difference between social sniffing and object sniffing in Nestin-WT animals, but no difference in *Anks1b* Nestin-Het mice as expected (**New Table S5**).

19) "In figure 3D, PPI is performed I cannot find the length of 'long delay'... In figure 3F, I cannot find how the preference for new is calculated."

We apologize for these omissions. Short-delay PPI has an inter-stimulus interval of 40 ms (**New Figure 3D**) and long-delay PPI has an interval of 200 ms (**New Figure S3E**). These details have been added to the revised Methods section.

20) "In figure 3G, the number of male and female mice should be informed. The number of each gender should be balanced to prevent biased results."

Numbers for males and females of each genotype have been added to **New Figure 3G** to show a balanced number of animals in each experiment. **New Figure 3, Figure S3,** and **Table S5** include a new cohort of 28 adult animals. As before, no behavioral results showed sex-dependent effects of genotype in 2-way ANOVA (**New Table S5**).

21) In figure 4, many interesting interactions were detected in quantitative proteome analysis. To strengthen molecular mechanistic insight, these interactions should be validated by Co-IP or Pull-down assay.

As suggested, we have performed co-immunoprecipitation experiments that confirm several interesting proteins in the AIDA-1 interactome identified by our proteomics experiment. In **New Figure 4D**, we show that IP of AIDA-1 shows interactions with ARF GTPase-activating protein 1 (Git1), Intersectin-1 (Itsn1), Adaptor Related Protein Complex 2 (Ap2a1/2), ARF1 GTPase-Activating Protein (Asap1), and SLIT-ROBO Rho GTPase Activating Protein 1 (Srgap2), which are proteins that regulate vesicle trafficking and synaptic function.

22) "In figure 1A, it would be better to indicate alternative transcription start site. Some deletions can still let the short transcripts made."

As suggested, we have indicated the two putative transcription start sites in **New Figure 1A**. Although the start site for shorter transcripts is downstream of all microdeletions identified, we show by Western blot and RT-qPCR that downstream isoforms and exons are similarly down-regulated in *ANKS1B* syndrome patients.

REVIEWERS' COMMENTS:

Reviewer #1 (Remarks to the Author):

OK - nice responses

Reviewer #2 (Remarks to the Author):

They almost fully covered my comments but I still have several remained concerns before publication.
1. In three chamber assay, ‘likelihood ratio effect test’ was considered for passing and failing, but I cannot understand the concept of this test. Why did you add this data?

2. Young HT mice show normal behavioral performance even though adult animals don’t. If so, I think there are little clue that AIDA-1 HT mice shows ‘neurodevelopmental’ defects… the only clue of that is the brain size measured at ‘adult’ stage. Even though pre-weaning stage is not affected by haploinsufficiency of AIDA-1, post-weaned, juvenile mice may have behavioral deficits. Juvenile play behaviors or juvenile 3-chamber test is likely to be necessary to support the title of this study.

3. In line 481, NDMAR should be NMDAR.

We thank the reviewers for their time and input. We are happy that Reviewer #1 was fully satisfied, and that Reviewer #2 was "almost fully" satisfied with our revisions. Below is a point-by-point response to the concerns raised by Reviewer #2.

Reviewer #2
They almost fully covered my comments but I still have several remained concerns before publication.
1. In three chamber assay, 'likelihood ratio effect test' was considered for passing and failing, but I cannot understand the concept of this test. Why did you add this data?

We added data from an additional mouse cohort, but did not perform any new behavioral tests. The new pass/fail representation (**Supplementary Figure 3D**) is simply a different way to present the deficit in social preference (**Figure 3C**). The likelihood ratio test is a goodness-of-fit statistical analysis used to determine how well the data fit a particular model. In this case, we used it to determine how likely the animal was to pass the test given its genotype, which is described in the Methods. In addition to the likelihood ratio test, Pearson's chi-square, and Fisher's exact test were also performed on the data, and they are presented in **Supplementary Data 5**. For all statistical tests, there was a significant effect of genotype on pass/fail likelihood (p<0.001).

2. Young HT mice show normal behavioral performance even though adult animals don't. If so, I think there are little clue that AIDA-1 HT mice shows 'neurodevelopmental' defects… the only clue of that is the brain size measured at 'adult' stage. Even though pre-weaning stage is not affected by haploinsufficiency of AIDA-1, post-weaned, juvenile mice may have behavioral deficits. Juvenile play behaviors or juvenile 3-chamber test is likely to be necessary to support the title of this study.

The title of our study does not refer to the mouse model, but to the novel human genetic disease caused by loss of *ANKS1B*. The cardinal features of *ANKS1B* haploinsufficiency syndrome are autism, ADHD, speech delays, and motor dyspraxia (**Table 1**), all of which fall under the diagnostic category of Neurodevelopmental Disorders in DSM-V (*Diagnostic and Statistical Manual of Mental Disorders*, https://doi.org/10.1176/appi.books.9780890425596.dsm01). Therefore, "neurodevelopmental" is the most precise descriptor for the spectrum of patient presentations in *ANKS1B* haploinsufficiency syndrome.

We have demonstrated that *Anks1b* Nestin-Het mice show most of the behavioral correlates for the neurodevelopmental diagnoses in patients. We acknowledge that this particular model does not replicate all human disorders at the ages and assays tested. This is a rather common caveat for animal models of disease due to many possible reasons, including developmental compensation, critical periods, and species differences. As indicated by the reviewer, the mice show reduced brain size (**Figure 2D**), a typical indicator of impaired neural development that should not be downplayed. Since these brains show no signs of neurodegeneration (**Supplementary Figure 2E**), we can think of no relevant causal mechanism other than altered development.

Finally, we have tested 18 distinct behavioral assays in our mouse model covering the spectrum of neurodevelopmental phenotypes observed in patients, and the additional behavioral experiments suggested by the reviewer would not yield any new information about how *ANKS1B* haploinsufficiency contributes to neurodevelopmental disorders.

3. In line 481, NDMAR should be NMDAR."

This has been corrected in the text.
